# ATLANTIS - Attractor Landscape Analysis Toolbox for Cell Fate Discovery and Reprogramming

**DOI:** 10.1038/s41598-018-22031-3

**Published:** 2018-02-23

**Authors:** Osama Shiraz Shah, Muhammad Faizyab Ali Chaudhary, Hira Anees Awan, Fizza Fatima, Zainab Arshad, Bibi Amina, Maria Ahmed, Hadia Hameed, Muhammad Furqan, Shareef Khalid, Amir Faisal, Safee Ullah Chaudhary

**Affiliations:** 1 Biomedical Informatics Research Laboratory, Department of Biology, Syed Babar Ali School of Science and Engineering, Lahore University of Management Sciences, Lahore, 54792 Pakistan; 2 Cancer Therapeutics Laboratory, Department of Biology, Syed Babar Ali School of Science and Engineering, Lahore University of Management Sciences, Lahore, 54792 Pakistan

## Abstract

Boolean modelling of biological networks is a well-established technique for abstracting dynamical biomolecular regulation in cells. Specifically, decoding linkages between salient regulatory network states and corresponding cell fate outcomes can help uncover pathological foundations of diseases such as cancer. Attractor landscape analysis is one such methodology which converts complex network behavior into a landscape of network states wherein each state is represented by propensity of its occurrence. Towards undertaking attractor landscape analysis of Boolean networks, we propose an Attractor Landscape Analysis Toolbox (ATLANTIS) for cell fate discovery, from biomolecular networks, and reprogramming upon network perturbation. ATLANTIS can be employed to perform both deterministic and probabilistic analyses. It has been validated by successfully reconstructing attractor landscapes from several published case studies followed by reprogramming of cell fates upon therapeutic treatment of network. Additionally, the biomolecular network of HCT-116 colorectal cancer cell line has been screened for therapeutic evaluation of drug-targets. Our results show agreement between therapeutic efficacies reported by ATLANTIS and the published literature. These case studies sufficiently highlight the *in silico* cell fate prediction and therapeutic screening potential of the toolbox. Lastly, ATLANTIS can also help guide single or combinatorial therapy responses towards reprogramming biomolecular networks to recover cell fates.

## Introduction

Biomolecular interplay at the subcellular level regulates cellular processes such as proliferation and apoptosis^1^. Aberrations in the regulatory control of these cellular processes can give rise to system-level disorders like cancer, diabetes and Alzheimer’s disease^2–5^. Specifically, in case of cancer, such system level abnormalities induced by dysregulated subcellular interplays have been termed as *Hallmarks of Cancer*^6,7^.These hallmarks synergize to irreversibly alter the developmental mode and consequence for a cell (i.e. *cell fate*)^7–9^. An integrative analysis of biomolecular pathways involved in modulation of critical cellular processes can therefore help predict cell fate outcomes^10–12^. Limited capacity of experimental protocols to simultaneously investigate multifactorial regulation of complex processes^13,14^ necessitates utilization of computational tools for predicting cell fates^11,15^.

Towards computational analysis of biological pathways, interactions between biomolecules are typically represented using directed graphs or networks. Boolean modelling paradigm has been widely employed as a suitable strategy for evaluating the system level outcomes of such biomolecular networks^16–19^. In this approach, interacting biomolecules are linked together into a network of nodes and edges (connecting two nodes). Each node has a discrete *on* or *off* state based on its activity while each edge carries a *weight* describing node interaction strength^15,20^. This strategy conveniently captures all possible node state combinations thus representing the overall network topology and function^15,20^. Since, cell fates are emergent properties of synergistic network states^21,22^, the mapping of Boolean network states to cell fate requires aggregation of related states. Such a grouping of associated states thus classifies the cell fate. Cell fate prediction^23–25^ using grouped gene regulation network states has been envisaged in *Waddington’s Epigenetic Landscape*^22,26,27^. *Attractors* are recurring stable network states having the highest likelihood of emergence^20^. Han *et al*. successfully employed this adaptation and quantified propensities (also termed *potential energies*) of each network state in a Yeast cell cycle network^23^. Later, Li *et al*. uncovered the Mexican hat landscape governing mammalian cell cycle’s progression through G1, S/G2 and M phases towards adaptation of various cell fates^28^. In another application of potential energy landscapes, Wang *et al*. investigated the temporal evolution and determination of stem cell differentiation process^29^. Recently, Cho *et al*. utilized attractor landscape analysis to develop a conceptual basis for the reversal of otherwise irreversible cellular fates like differentiation, cellular aging and tumorigenesis^24^.

A wider application of attractor landscape modelling for cell fate determination and reprogramming is hampered by the lack of an integrative modelling and analysis pipeline. Previously, several tools have been developed for construction^30,31^, visualization^32,33^ and analyses^34,35^ of logical networks. From amongst them, the leading tools for computing attractor states include BoolNet^36^, The Cell Collective^37^ and CellNetAnalyzer^38^. However, these tools fail to associate biologically relevant network states with emergent cell fates. It is the lack of this functionality which further impedes cell fate reprogramming in light of molecular cues from attractor states. To address this need, we propose *ATLANTIS*, a MATLAB toolbox for determining and reprogramming cell fates using attractor landscape analysis of biomolecular networks. ATLANTIS allows its users to conveniently create, modify, visualize and analyze Boolean networks. Deterministic (*closed*) or stochastic (*noisy*) modalities of network analyses can be followed by cell fate association to the emergent network states. Users can then also reprogram these cell fates by systematically perturbing underlying biomolecular networks. A list of these features along with a comparison of ATLANTIS with existing tools is provided in Supplementary Information (Comparison of Features - ATLANTIS vs. Other Tools).

To validate cell fate prediction capability of ATLANTIS, three different case studies including (i) predicting G1 cell fate in budding yeast^23^, (ii) p53 mediated apoptosis in MCF-7 breast adenocarcinoma cell lines^19^, and (iii) evolution of cell fate landscape with accumulation of mutations in colorectal tumorigenesis^24^ were undertaken. ATLANTIS successfully classified the cell fates in each case study i.e. stationary G1 phase (case study 1), p53-induced apoptosis in MCF7 cell lines (case study 2), and tumorigenesis in colorectal adenocarcinoma (CRC) (case study 3). We also used ATLANTIS to study the changes in cell fate outcomes of HCT-116 cell network upon inhibition of various target nodes. The efficacious drug target nodes were identified based on the impact of their inhibition on basin sizes of cell fates including normal and abnormal proliferation, metastasis, cell cycle arrest and apoptosis attractors. Inhibitors targeting ERK, EGFR, MEK, AKT, PI3K, RAF nodes and P53-MDM2 interaction reduced normal and abnormal proliferation, and metastasis. On the other hand, this inhibition increased cell cycle arrest and apoptosis. We also compared the GI_50_ values of inhibitors targeting the aforementioned nodes to various cell fates and these values (i.e. basin sizes) conformed to the known efficaciousness of these drugs. These cell fate predictions made using *in silico* screening in ATLANTIS were in accordance with the experimental data.

Such cell fate determination capability of ATLANTIS can be employed in virtual drug screening where subsequent drug perturbations allow for switching between different cell fates. Moreover, latent cell fates can also be deciphered by dynamically converging the system into specific network states (also called steady states). In conclusion, the aforementioned cell fate determination case studies signify the utility of the proposed toolbox in building a better understanding of complex multifactorial cellular processes.

## Results

### Overview of ATLANTIS Toolbox – Graphical User Interface and Analysis Pipeline

**Figure 1:**
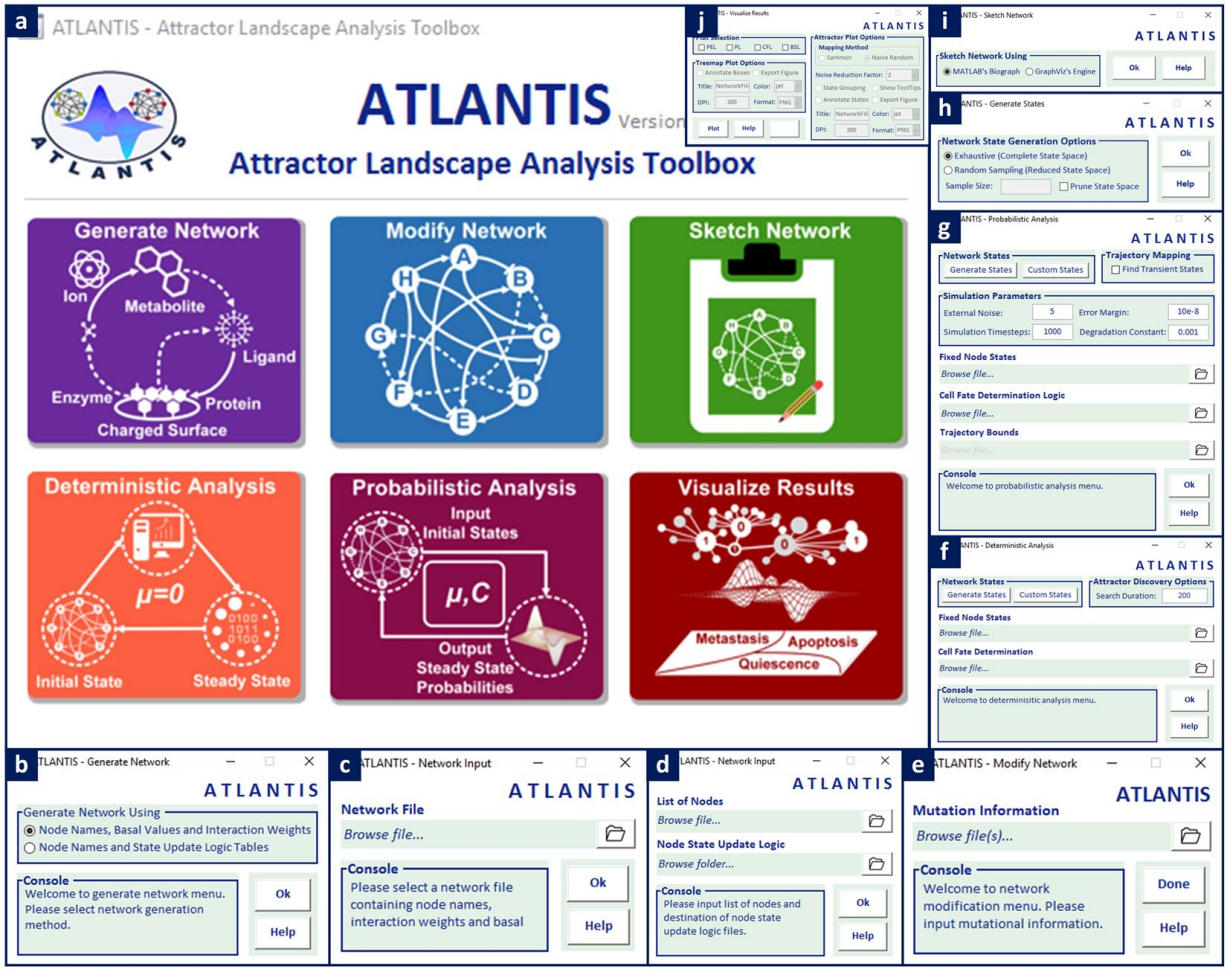
Graphical User Interfaces of ATLANTIS Toolbox. (**a**) Main window of the toolbox for network input, modification, analysis and visualization, (**b**) network file input dialog for loading network data from text or Microsoft Excel® files, (**c**) interaction weights based network generation dialog, (**d**) rules-based network generation dialog, (**e**) network file modification dialog for editing node states and basal values as well as edge interaction weights, (**f**) deterministic analysis dialog, (**g**) probabilistic analysis dialog, (**h**) *de novo* network states generation dialog for use with deterministic and probabilistic analyses, (**i**) network sketching dialog, (**j**) visualize results dialog for plotting attractor and cell fate landscapes.

ATLANTIS is an open source software (see Supplementary Availability) for attractor landscape analysis of biomolecular networks towards determining cell fates. The proposed toolbox (Fig. 1a) is developed using the popular scientific computing platform MATLAB (R2016a)^39^. ATLANTIS admits Boolean network generation from node states and edge weights, logic rules-based node updates, network modification via node and edge knock-down, cell fate determination using user-defined logic and network visualization using MATLAB Biograph object^40^ and Graphviz^41^ (Fig. 1b–e,i). The analysis pipeline includes both deterministic as well as probabilistic analyses of user-defined networks (Fig. 1f–h). Attractors can be visualized in the form of attractor landscapes whereas the cell fates associated with each attractor can be viewed as cell fate landscapes (Fig. 1j).

**Figure 2:**
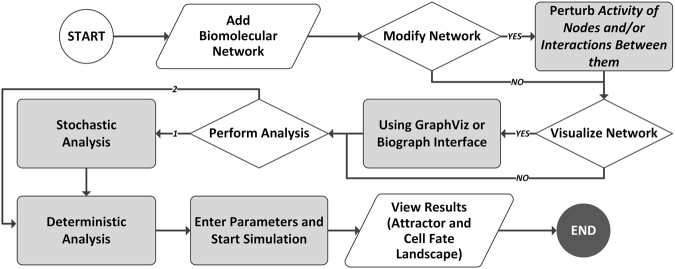
Workflow of ATLANTIS. The salient steps in the attractor landscape construction and cell fate prediction process include data input, model analysis and result visualization.

ATLANTIS pipeline (Fig. 2) is initialized with input of biomolecular information. This information includes node data (initial state, basal value, state update logic), interaction data (type and weight), degradation constant and noise level (see Supplementary Information - Step by Step). Users can also modify the node and interaction data or perturb it before onward analysis. Network visualization can be performed using Graphviz^41^ or Biograph. ATLANTIS provides deterministic and stochastic analyses of Boolean networks. Analysis results in the form of attractors and cell fates can be visualized as landscapes and treemaps, respectively (see Supplementary Information - Worked Examples).

### Case Study 1 – Decoding Yeast Cell Cycle Progression using Attractor Landscape Analysis

**Figure 3:**
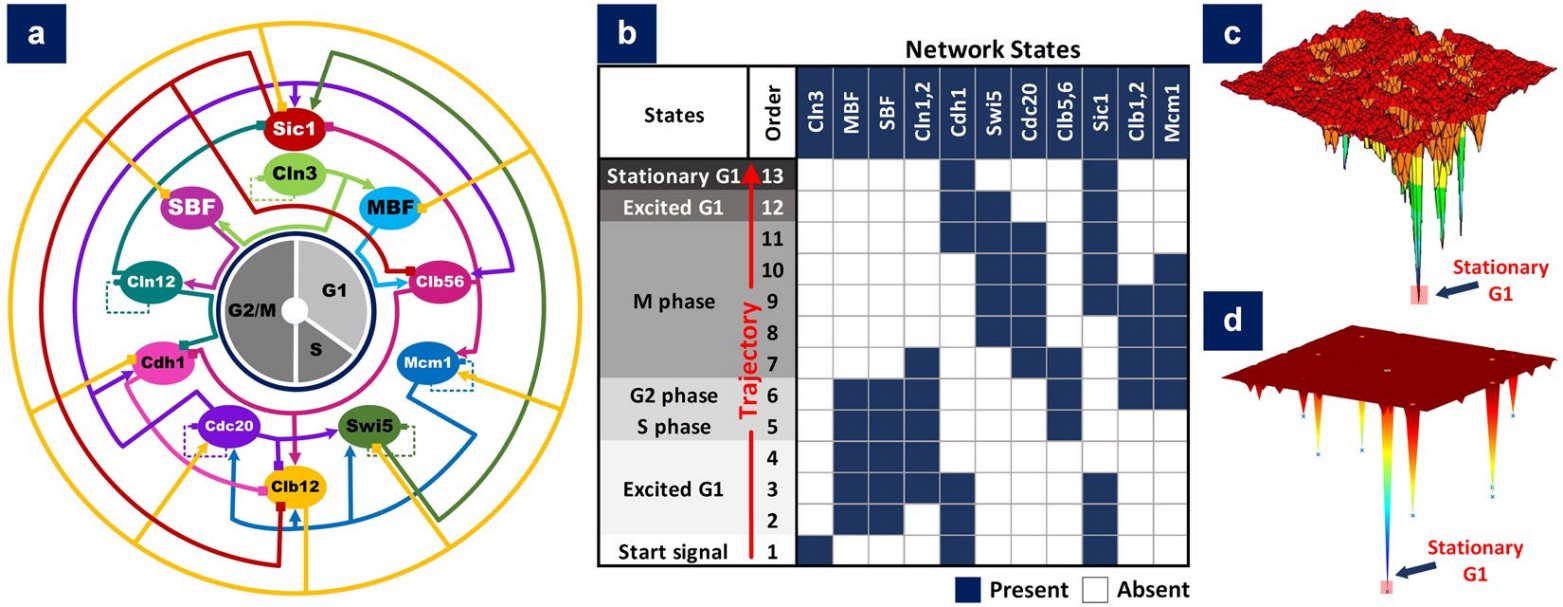
Decoding Yeast Cell Cycle Progression. (**a**) 11 node yeast cell cycle network adapted from Han *et al*., (**b**) yeast cell cycle trajectory order (1 → 13) during cell fate adoption from start signal to stationary G1 phase, (**c**) attractor landscape constructed by Han *et al*., (**d**) attractor landscape constructed using ATLANTIS toolbox.

To evaluate the accuracy of cell fate predictions made using ATLANTIS, we reconstructed a study by Han *et al*. for eliciting phase trajectory of yeast cell cycle network^23^. For that, we leveraged the transient states identification capability of probabilistic analysis (PA) pipeline. First, the yeast cell cycle network (Fig. 3a) was loaded into ATLANTIS which was followed by PA for attractor landscape construction. For establishing the cell cycle trajectory adapted by yeast cells, progressive onset of various cell cycle phases was studied starting from a “*start signal state*”. The trajectory concluded at the stationary G1 attractor after passing through several intermediary cell cycle phases (Fig. 3b). Each phase was represented in steady state by one or more network state(s) (details in Supplementary Results – Case Study 1). Our results showed that the probability (a measure of stability) of each successive state in cell cycle trajectory increased until the stationary G1 attractor state was achieved. This could be explained by the fact that for achieving steady state, biological systems increasingly adopt stable states with higher probabilities as compared to the ones having lower probabilities. Importantly, the order of onset of each phase conformed to the actual biological path^42^ in yeast cells during cell cycle progression (i.e. Start Signal → Synthesis (S) → Gap 2 (G2) → Mitosis (M) → Gap 1 (G1)) (Fig. 3b). This trajectory was in agreement with the predictions made by Han *et al*.^23^. The attractor landscape reported earlier by Han *et al*. (Fig. 3c) was reproduced by ATLANTIS (Fig. 3d) (runtime of 7 seconds, Supplementary Information – Performance Analysis of ATLANTIS).

To further confirm the emergence of Stationary G1 state as the most likely steady state attractor, we also performed deterministic analysis (DA) and compared the attractor propensities with BoolNet^36^. ATLANTIS took 2 seconds to perform the analysis (see Supplementary Information – Performance Analysis of ATLANTIS) and reported a total of seven attractor states (see Supplementary Results – Table S2), wherein DA successfully ascertained the results generated by PA. Among these, stationary G1 attractor state had the largest basin size (~0.85), suggesting that 85% of the yeast cell population would adopt this state. The remaining attractor states were not part of the predicted cell cycle trajectory and can be described as “off-pathway traps” as indicated earlier by Han *et al*.^23^ in their work.

### Case Study 2 – Reprogramming P53-mediated Apoptosis in MCF-7 Breast Cancer Cell Lines

**Figure 4:**
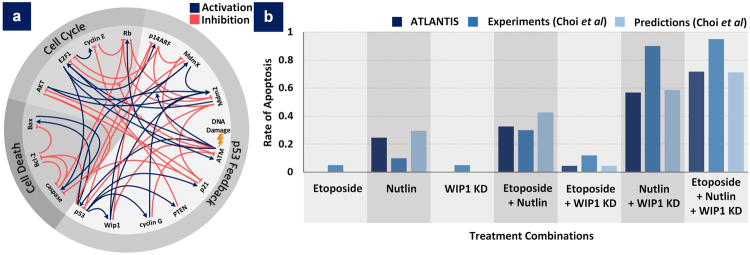
Evaluation of Combinatorial Therapeutic Efficacy to enhance P53-mediated Apoptosis in MCF-7 Breast Cancer Cell Lines. (**a**) P53 signaling network adapted from Choi *et al*.^19^. (**b**) Individual and combinatorial effects of Etoposide (E), Nutlin (N) and WIP1 knock-down (W) on P53-mediated apoptosis in MCF-7 cells from Choi *et al*. (experiments and predictions) and ATLANTIS.

ATLANTIS can be employed to study reprogramming of cell fates via selective modifications of biomolecular networks guided by attractor landscape analyses. Such reprogramming of cell fate by specific adjustments of attractor states can be useful in developing combinatorial therapeutic leads. In one such study, Choi *et al*. employed attractor landscape analysis to tailor a combinatorial drug screening strategy for enhancing P53 mediated apoptosis in MCF-7 breast cancer cell lines^19^. We setup ATLANTIS to perform *in silico* drug screen on the MCF-7 P53-mediated apoptosis network proposed by Choi *et al*. (Fig. 4a) using deterministic analysis (DA). Here, we used DA to efficiently identify both point and cyclic attractors. The drugs used to treat the network included Etoposide (E) and Nutlin (N) along with WIP1 knock-down (W). Etoposide causes DNA damage^43^, Nutlin inhibits MDM2 and P53 complex formation^44^, and WIP1 knock down prevents dephosphorylation and inactivation of P53 and ATM by WIP1^19,45^. Additionally, combinations of these drugs (i.e. E + N, E + W, N + W and E + N + W) were also included in the screen. DA was performed to compute the basin sizes of cell death (Fig. 4b) and other emergent attractor states (see Supplementary Table S3). First, MCF-7 network was simulated in normal condition i.e. without any therapeutic intervention. A single proliferation attractor was observed for control, with a basin size of 1.0 (see Supplementary Figure S9). This result indicated that MCF-7 cells are in a proliferative state in normal conditions. Next, the individual effects of E, N and W were studied on MCF-7 network. The predicted rate of apoptosis for each treatment was compared with the experiments performed by Choi *et al*.^19^ (Fig. 4b). ATLANTIS predicted no apoptosis after treatment with E and W as compared to 5% apoptosis in experimental observations. For N treatment, ~25% apoptosis was observed versus the experimental rate of 10%. For evaluating the combinatorial effects of these drugs, treatment with various combinations of these drugs was undertaken. Drug combinations along with the ATLANTIS-predicted versus experimentally observed rates of apoptosis are as follows: E + N, ~33% versus 30%, E + W, ~5% versus 12%, N + W, ~57% versus 90%, and E + N + W, ~72% versus 95%, respectively. Summarily, DA pipeline successfully reported the point and cyclic attractor states, as reported by Choi *et al*., in under 12 seconds (see Supplementary Information – Performance Analysis of ATLANTIS).

We also compared these basin size-based cell fate predictions from ATLANTIS with BoolNet (see Supplementary Table S3). Each attractor type as predicted by ATLANTIS was consistent with the outcome from BoolNet having comparable basin sizes. Lastly, to validate the robustness of the computed attractors in a noise-tolerant setting, we also performed PA to simulate the aforementioned network. For the highly efficacious combination of E + N + W, ATLANTIS elicited 3 point attractors for cell senescence and 2 point attractors for cell death (see Supplementary Figure S10). These attractor states also corresponded well with the findings of Choi *et al*. The runtimes incurred were 2 seconds and 206 seconds on server configuration for heuristic and exhaustive PA, respectively (see Supplementary Information – Performance Analysis of ATLANTIS). Taken together, cell fate predictions and their post-therapeutic reprogramming as predicted by ATLANTIS were comparable to those reported by BoolNet and Choi *et al*.

### Case Study 3 – Investigating the Evolution of Cell Fate Landscape during Colorectal Tumorigenesis

**Figure 5:**
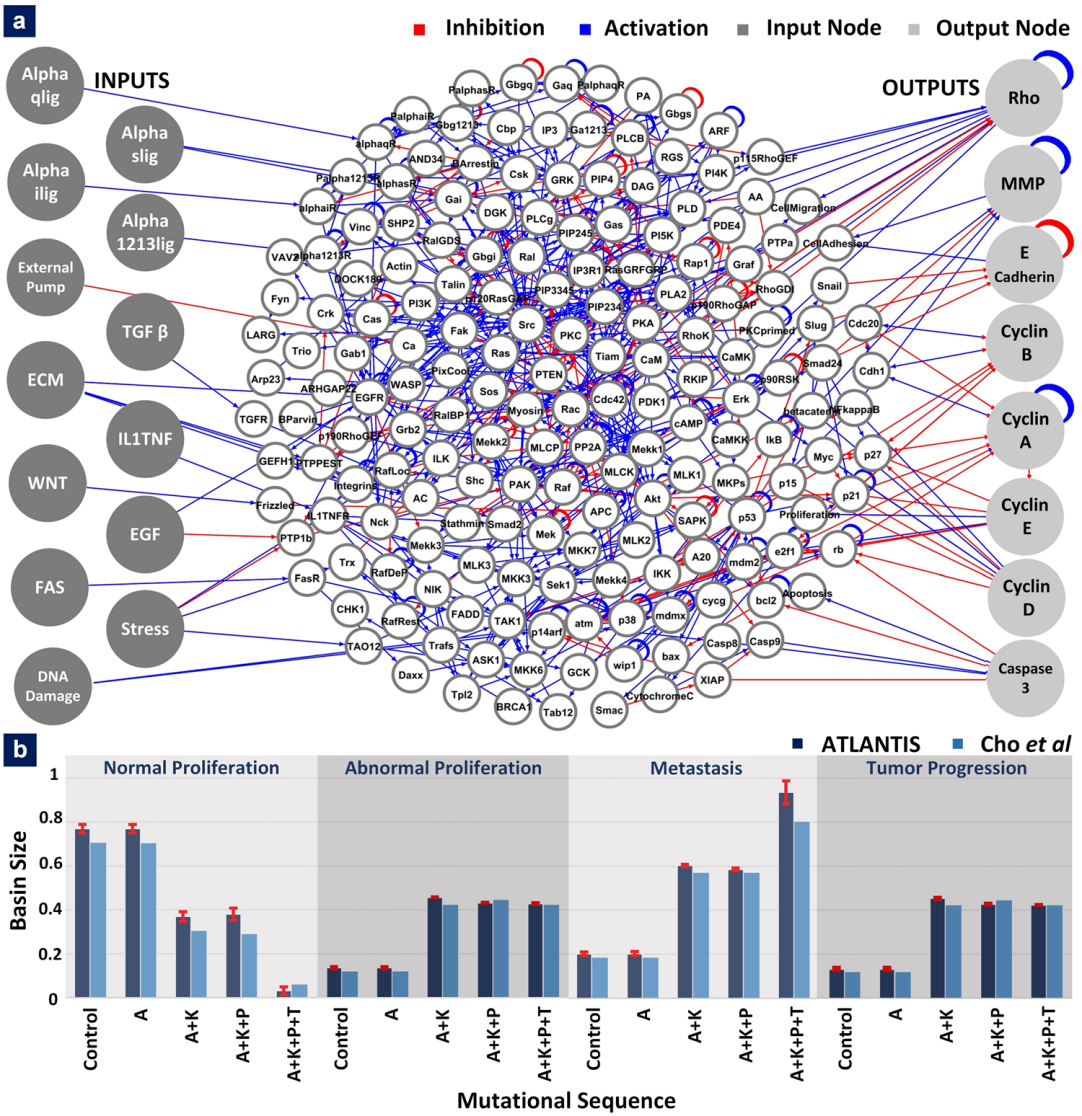
Temporal Evolution of Cell Fate Landscape during Colorectal Tumorigenesis. (**a**) 201-node human signaling network with 13 input nodes and 8 output nodes, (**b**) Evolution of cell fate landscape during colorectal tumorigenesis as predicted by ATLANTIS using the inferred logic. The driver mutations were added in the following order; APC (A), KRAS (K), PTEN (P) and TP53 (T) mutations.

In a recent study^24^, Cho *et al*. investigated the role of progressive mutations in human colorectal tumorigenesis using attractor landscape analysis. Here, we have adapted the 201-node network constructed by Cho *et al*. (Fig. 5a) and investigated temporal evolution of cell fate landscape during tumorigenesis. We use DA for tractably computing the large network steady states. Node state update rules and cell fate determination logic were inferred for performing DA of the network. Driver mutations including APC (Adenomatous Polyposis Coli)^46^, KRAS (Kirsten Rat Sarcoma viral oncogene homolog)^47^, PTEN (Phosphatase and Tensin homolog)^48^ and TP53 (Tumor Protein p53)^49^ were successively incorporated into the network. ATLANTIS took less than an hour to analyze 10,000 randomly generated states (see Supplementary Information – Performance Analysis of ATLANTIS). Resultant attractor landscapes were characterized and associated with cell fates including normal and abnormal proliferation, metastasis and tumor progression.

Our results show that basin size of attractors representing normal proliferation declined with accumulation of sequential driver mutations. The basin sizes for normal proliferation were observed to be 0.79, 0.79, 0.39, 0.41 and 0.013 for control, APC, APC + KRAS, APC + KRAS + PTEN and APC + KRAS + PTEN + TP53, respectively (see Supplementary Figures S11–15). The results conformed to the trend reported by Cho *et al*. in their work. Next, we computed the basin sizes of abnormal proliferation, metastasis and tumor progression attractors for control as well as successively mutated APC, KRAS, PTEN and TP53 (Fig. 5b, top row). The ATLANTIS-predicted increase in abnormal cell fates with accumulation of mutations was in agreement with the Cho *et al*.

### Case Study 4 – Discovering Cell Fate Outcomes in HCT-116 Cells and their Reprogramming upon Inhibition of Various Nodes in its Network

*In silico* analyses can assist in high-throughput screening of therapy-induced cell fate reprogramming, at a much lower cost and in a shorter time. ATLANTIS provides this capability by working off biomolecular network information, introducing changes (or mutations) in network and by perturbing the activity of nodes and/or interactions between nodes and associating steady states with cell fates. To demonstrate this, we employed ATLANTIS to investigate the adaptation of various cell fates by HCT-116 cells after inhibition of 16 drug target nodes (proteins in the network). The information on node-inhibitor pairs was taken from Genomics of Drug Sensitivity in Cancer (GDSC)^50^. Additionally, HCT-116 characteristic mutations were incorporated into the 201-node network (Fig. 5a) developed by Cho *et al*.^24^ (see Methods and Materials). The resultant large-scale network was deterministically analyzed followed by identification of attractors at steady state in under 45 minutes (see Supplementary Information – Performance Analysis of ATLANTIS). Attractor characterization was followed by their association with cell fates including normal proliferation, abnormal proliferation, metastasis, cell cycle arrest and apoptosis.

**Figure 6:**
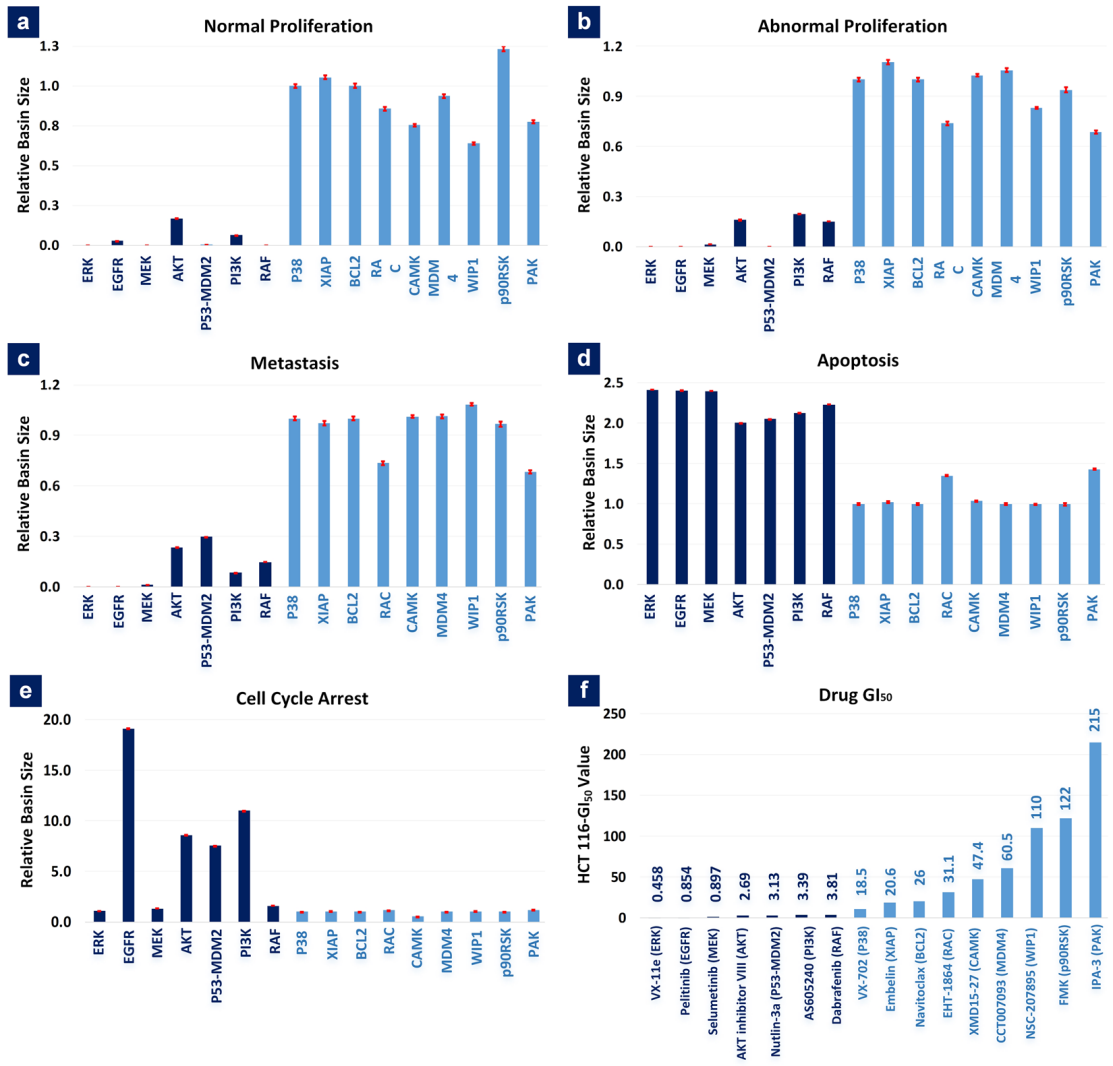
*In silico* Drug Screening in ATLANTIS. Relative basin sizes of (**a**) normal proliferation, (**b**) abnormal proliferation, (**c**) metastasis, (**d**) apoptosis and (**e**) cell cycle arrest after inhibition of 16 different drug targets in HCT-116 representative biomolecular network. (**f**) GI_50_ values of cells upon inhibition of various drug targets. The x-axis in 6a-e represents various drug targets that were inhibited and 6 f shows the drug-target combinations. The y-axis in 6a-e represents relative basin sizes for each cell fate. The basin size values are relative to the propensity of the cell fate in the untreated HCT-116 cell network (control).

Towards *in silico* screening of drug efficacy in HCT-116 cells, we investigated the drug-targeting effect of 16 different nodes on five cell fates. ATLANTIS predicted that significant amount of apoptosis would be induced by inhibition of ERK, EGFR, MEK, AKT, P53-MDM2 interaction, PI3K, and RAF in HCT-116 cells (Fig. 6e). Alongside, a reduction was observed in the basin size of normal proliferation, abnormal proliferation and metastasis attractors after corresponding target node inhibition (Fig. 6a–c). Inhibition of targets that induced apoptosis, also induced substantial cell cycle arrest with the exception of MAPK signaling inhibition (i.e. ERK, MEK and RAF) (Fig. 6d). The lowered cell cycle arrest can be explained by the loss of positive regulation of P21 production^51^, which is induced by MAPK pathway through ERK signaling^52–54^.

Next, the efficacy of inhibiting each target was computed by employing basin sizes for the aforementioned cell fates. Inhibition of targets exhibiting high-efficacy reduced the basin size of normal proliferation (Fig. 6a), abnormal proliferation (Fig. 6b) and metastasis (Fig. 6c) attractors. Moreover, an increase in basin size of cell cycle arrest (Fig. 6d) (with the exception of ERK, MEK and RAF inhibitions) and apoptosis (Fig. 6e) was observed upon inhibition of these targets. Importantly, these findings were in accordance with the GI_50_ values of each inhibitor corresponding to the selected target. The drugs that were predicted to be efficacious had low GI_50_ values (less than 3.81, Fig. 6f (shown in dark blue)) as compared to high GI_50_ values (greater than 18.5, Fig. 6f (shown in light blue)) for the rest (Fig. 6f). In conclusion, ATLANTIS was employed for predicting the drug-induced reprogramming of HCT-116 cell fates and the results were in line with the experimental observations.

## Discussion

Computational modelling and simulation of biomolecular networks has provided significant assistance in decoding regulatory mechanisms underpinning cellular life. Systemic analyses of these models can further help identify the key biomolecular regulators within each network. *Attractor landscape* analysis is one such approach which computes the steady states of networks and their corresponding propensities towards determining high-propensity *attractor* and low-propensity *transient* states. Together, the transient states converging into an attractor state form a *basin of attraction* within the overall attractor landscape. Several recent studies have employed attractor landscape analysis for deciphering emergent cellular behavior such as the cell fate^19,23,24^. This approach has also been applied to measure and modulate the therapeutic response of drug targets by perturbing the underlying networks^55,56^. However, lack of a computational pipeline for attractor landscape analysis impedes a wider adaptation of this effective methodology for decoding cell fates from biomolecular networks.

For addressing this need, we have proposed ATLANTIS, a publicly available MATLAB toolbox for attractor landscape analysis towards cell fate discovery and reprogramming (Fig. 1). ATLANTIS enhances the state-of-the-art attractor analysis of Boolean networks by provision of an intuitive tool for landscape construction and analysis. A feature-wise comparison of ATLANTIS and similar existing tools depicts how the proposed pipeline plugs the critical gap in tools for attractor landscape analysis and cell fate determination (see Supplementary Information – Tool Comparison). To demonstrate the capabilities and potential of the toolbox, four case studies were improvised using published data. ATLANTIS successfully reproduced the cell cycle trajectory in yeast cells^23^ (Fig. 3), the p53-mediated apoptosis in MCF-7 cell line^19^ (Fig. 4), and multi-stage colorectal tumorigenesis^24^ (Fig. 5). ATLANTIS was then also shown to identify efficacious drug-targets in HCT-116 cells by evaluating the impact of systemic node inhibitions on the propensities of various cell fates (Fig. 6). Prospectively, ATLANTIS users can also employ deterministic as well as probabilistic analysis (PA) of custom state space with fixed nodes for identification of network modules governing disease progression^24,57^. Follow-up incorporation of novel mutations by node or edge perturbations can also help predict their potential roles in disease^57^. Together, these two capabilities can be employed for *in silico* network-based drug screening and scoring strategies. Incorporation of expression data from patients can further guide such drug screening approaches towards precision medicine and personalized therapeutics^19,24,57–60^. Additionally, steady-state attractors defining tumor cell fates can also be studied in light of their transient progenitor states thereby providing cues for cell fate reprogramming^24,56^.

A major impediment in realization of the aforementioned use cases is the forbidding computational runtime associated with analysis of large state spaces. In this context, towards ascertaining the relevance of ATLANTIS, we have analyzed the runtime performance of the toolbox (see Supplementary Information – D. Performance Analysis of ATLANTIS). The computational cost determined was then compared with leading tools such as BoolNet, BooleSim and GINsim. Alongside, the effect of (i) increasing network sizes (11, 16 and 201 nodes), (ii) state-space projection algorithms (Sammon and Naïve mapping), and (iii) landscape visualization on toolbox performance have also been benchmarked. For features common to ATLANTIS and the aforementioned tools, the runtimes were found to be comparable. Importantly, the runtime of novel attractor landscape analyses was benchmarked and found to be nominal as well. Visualization of large networks using Graphviz is known to incur a large runtime cost. Since ATLANTIS employs Graphviz for this purpose, we have also provided additional support for sketching large networks through Biographs. Moreover, ATLANTIS-generated DOT files can also be used with Gephi^61^ for visualization and manipulation of networks.

In terms of efficiency of the deterministic analysis (DA) pipeline, ATLANTIS rules-based DA (~ 57 minutes for 10,000 randomly generated states) improves upon BoolNet (failed to compute) and BooleSim (updates a single state only). For exhaustive PA, however, a considerably large system memory is required by ATLANTIS. For addressing this issue, we have implemented a heuristic PA pipeline which works seamlessly on low-memory as well as large-memory systems. However, the heuristic analysis cannot elicit state-transition trajectories for attractor states. For that, exhaustive analysis becomes indispensable. Similarly, Sammon transformation takes a long time to cluster and map the high-dimensional network state-space onto a Cartesian plane. To facilitate this, we have additionally implemented a Naïve mapping strategy that is scalable to large networks. However, Naïve mapping cannot maintain the spatial relationship between various steady-states thereby creating a visual discrepancy in attractor positioning within the landscape. Lastly, ATLANTIS toolbox requires a MATLAB license. To accommodate users without MATLAB, the toolbox has also been packaged as an executable file (see Supplementary Information - Availability). However, further development of ATLANTIS code base still necessitates a MATLAB license.

In conclusion, the proposed attractor landscape analysis toolbox has been demonstrated to provide cell fate prediction and reprogramming capability. The novel toolbox offers to translate biomolecular networks to corresponding cell fates which can assist in drug-sensitivity analysis for applications in complex diseases such as cancer and diabetes.

## Methods and Materials

### ATLANTIS Toolbox - Development of Graphical User Interfaces and Analysis Pipeline

ATLANTIS was developed using MATLAB (R2016a)^39^, a widely employed scientific computing platform. The source code was designed following the Object-oriented programming (OOP)^62^ paradigm for an intuitive data abstraction, object reuse and code scalability. Alongside, a set of interactive graphical user interfaces (GUI) has been provided for creating, modifying and undertaking analysis of Boolean networks. MATLAB GUIDE (GUI Development Environment)^39^ was employed to construct each GUI figure. Figure files (‘.fig’) for each GUI have been provided along with the source code files (‘.m’) on the GitHub repository (see Supplementary Information IV-Availability).

ATLANTIS requires formatted flat text (‘.txt’) or Comma Separated Files (‘.csv’) data files containing network information for defining and modifying networks (see Supplementary Methods – Data Preparation). Networks modification can be also performed by providing mutational information as ‘.txt’ or ‘.csv’ formatted files (see Supplementary Methods – Data Preparation). For visualizing user-provided network data, Graphviz, a popular graph visualization application programming interface (API)^41^, was integrated into ATLANTIS. Graphviz API works off graph description language (.dot) files containing network visualization data provided by the user^63^. Additionally, for sketching large networks, support for MATLAB’s Biograph object^64^ has also been provided.

ATLANTIS supports analysis of biomolecular networks modelled using Boolean approach (see Supplementary Methods – Boolean Modelling of Biomolecular Networks). Node states, node interactions, node basal expressions and perturbation (or noise) are used in defining network models. The network analysis pipeline in ATLANTIS has been implemented using both (i) deterministic and (ii) probabilistic modelling approaches. The deterministic analysis (DA) pipeline was developed for analyses of closed systems wherein networks are not subjected to external perturbations or noise, *µ*. A node state update rule based on connectivity of the interacting nodes was used to simulate network state dynamics (see Supplementary Methods – Deterministic Analysis). For incorporating specific network regulation rules into deterministic analyses, network states were updated using node update rules from within the rules space. Flat text files with node input-output logics act as input for the rules-based deterministic analyses (see Supplementary Methods – Deterministic Analysis). Towards incorporating intrinsic signaling perturbations requisite for an open system, a probabilistic analysis (PA) pipeline was also developed. For that, state transition probabilities were computed by incorporating random noise, *µ*, into the state update rule (see Supplementary Methods – Probabilistic Analysis). An additional term, degradation constant, *c*, was included in the rule to control the activity of a node in a noisy environment by determining its self-degradation rate in the absence of regulation (see Supplementary Methods – Probabilistic Analysis). A master equation was then used to compute steady state probabilities^65^. Once a network attains its steady state using either DA or PA, most probable network states are termed as attractors (*point* or *cyclic*). The non-attractor “transient states” guide the evolution of low-energy attractor states thereby making up kinetic paths or trajectories. ATLANTIS allows users to extract the trajectory taken up by a network in going from an initial network state to the terminal attractor state *via* these intermediary transient states. These attractor states were projected into a two-dimensional space using Naïve or *Sammon* mapping^66^. The transformed network states along with relative frequency of each state were used to construct the global attractor landscape (see Supplementary Methods – Plotting Attractor Landscapes).

### Experimental Data for Case Studies

ATLANTIS uses highest likelihood network states to determine cell fates followed by construction of attractor or cell fate landscapes. To validate the cell fate prediction capability, published data from four different case studies was employed. For case study 1, network information including nodes, interaction weights and physiological state trajectories of yeast cell cycle progression towards G1 cell fate were obtained from Han *et al*.^23^ (see Supplementary Data – Case Study 1). For case study 2, network information including nodes, their basal values, interaction weights and cell fate determination logic was adapted from Choi *et al*.^19^ (see Supplementary Data – Case Study 2). For case study 3, network information including nodes, initial states, input and output nodes, node activity update logic and cell fate determination logic was obtained from Cho *et al*.^24^ and used for preliminary cell fate analysis (see Supplementary Data – Case Study 3). ‘Normal Proliferation’ (NP), ‘Abnormal Proliferation’ (AP), ‘Metastasis’ (M) and ‘Tumor Progression’ (TP) were further investigated for showing the shift from normalcy (NP) to malignancy (AP, M and TP) (Fig. 5). TP was defined as the concurrent occurrence of AP and M. Lastly, in case study 4, cell fate predictions from ATLANTIS were validated by performing an *in silico* screen on HCT-116 colon cancer cell line representative network. In this study, efficacies of 16 drug targets (proteins present in the network) were predicted. HCT-116 cell line representative network was made by introducing mutations specific to this cell line reported in COSMIC database by Sanger Institute^67^ (see Supplementary Methods –Table S1). Relative drug sensitivity data for this cell lines was acquired from Genomics of Drug Sensitivity in Cancer (GDSC)^50^.

### Validation of the Toolbox

Four different case studies were selected for validating ATLANTIS. Networks from each case study were reconstructed followed by cell fate determination and its reprogramming.

In case study 1, the state trajectory of budding yeast’s cell cycle progression, identified by Han *et al*.^23^, was compared using ATLANTIS (PA, at a noise value, ${\rm{\mu }}=5$ and degradation constant,*c* = 0.01). The model was then also analyzed by BoolNet - a Boolean network construction and analysis package^36^ and its results were compared with ATLANTIS using DA. The results were then qualitatively compared using a potential energy landscape.

For case study 2, the p53-mediated apoptotic network^19^ was analyzed by performing DA and the results were compared with BoolNet^36^. PA was then performed to find steady state probabilities which were used to construct the attractor landscapes. Apoptotic effects of treatments with Etoposide, Nutlin and WIP1-siRNA along with their combinations on MCF-7 cell-line were simulated using ATLANTIS. Basin size ratios of resulting cell death attractors were used to quantify the rate of apoptosis. These results were then compared with the experimental rates of apoptosis. The effects of Etoposide and WIP1 knock-down were incorporated by changing the basal expression of ATM to 1, and WIP1 to 0. The basal expression of Wip1 node was set to a large negative number resulting in permanent “off” (0) state. The ‘on’ state of ATM represents the increase in ATM levels in response to DNA damage caused by Etoposide. WIP1’s ‘off’ state represents low levels of active Wip1 after siRNA mediated knock-down. To capture the effect of Nutlin, the interaction from MDM2 to p53 in the MCF7 network was removed resulting in p53 node state being ‘on’ (1) even in presence of MDM2.

In case study 3, we evaluated the large-scale network analysis capability of ATLANTIS. A 201-node network, built by Cho *et al*.^24^ was analyzed using DA. A total of three custom states were generated. The first state space was built by using the network state list from the original study. The other two randomly sampled state spaces (with 10,000 states per sample) were generated by using ATLANTIS. The resulting sets of three state-spaces were analyzed and the results were averaged. We followed it up by evaluation of the impact of sequential mutations in APC, KRAS, PTEN and TP53 on normal and abnormal proliferation, metastasis and tumor progression. This was done by setting the node states of APC, KRAS, PTEN and TP53 to 0, 1, 0 and 0 in order, respectively. Basin sizes representing normal proliferation, abnormal proliferation, metastasis and tumor progression were calculated using rules-based DA.

In case study 4, we highlighted drug screening capability of ATLANTIS by predicting the effects of various node/link inhibitions in HCT-116 colon carcinoma cell network. We characterized the 201-node network constructed by Cho *et al*. to make it reflective of the HCT-116 cells. This was done by setting the node states of both RAS and PI3K to 1 (see Supplementary Methods - HCT-116 Characteristic Mutations). Next, we performed DA on the modified network towards predicting the efficacy of each drug-target combination.

## Electronic supplementary material

Supplementary Information

Case Study 1

Case Study 2

Case Study 3

Case Study 4

Overall runtimes for case studies 1 to 4

Individual Runtimes of Salient Features in ATLANTIS

Effect of Network Size on Analysis Runtimes

ATLANTIS MATLAB Dependencies
