# Supplementary material for: ATLANTIS - Attractor Landscape Analysis Toolbox for Cell Fate Discovery and Reprogramming
Source: Sci Rep. 2018 Feb 23;8:3554. doi: 10.1038/s41598-018-22031-3 (PMC5824948; doi:10.1038/s41598-018-22031-3)
Supplement: Supplementary file 1 — Supplementary Information [file 41598_2018_22031_MOESM1_ESM.pdf]

Supplementary Information

ATLANTIS - Attractor Landscape Analysis Toolbox for Cell Fate Discovery and Reprogramming

Osama Shiraz Shah[1], Muhammad Faizyab Ali Chaudhary[1], Hira Anees Awan[1], Fizza Fatima[1], Zainab Arshad[1], Bibi Amina[1], Maria Ahmed[1], Hadia Hameed[1], Muhammad Furqan[2], Shareef Khalid[1], Amir Faisal[2], Safee Ullah Chaudhary[1,] *

[1] Biomedical Informatics Research Laboratory, Department of Biology, Syed Babar Ali School of Science and Engineering, Lahore University of Management Sciences, Lahore, Pakistan.

[2] Cancer Therapeutics Laboratory, Department of Biology, Syed Babar Ali School of Science and Engineering, Lahore University of Management Sciences, Lahore, Pakistan.

*Corresponding author
E-mail: safee.ullah.chaudhary@gmail.com, **Phone:** +92-42-350-8352, **Fax:** +92-42-350-4310, **Web:** biolabs.lums.edu.pk/birl

Contents

A. Supplementary Methods 2

1. Data Preparation for use with ATLANTIS 2

2. Boolean Modelling of Biomolecular Networks 4

3. State Transitions and Network Dynamics 4

4. Deterministic Analysis of Boolean Networks 5

5. Probabilistic Analysis of Boolean Networks 7

6. Plotting Attractor and Cell Fate Landscape 9

7. HCT-116 Characteristic Mutations 10

B. Supplementary Results 11

1. Case Study 1 – Decoding Yeast Cell Cycle Progression using Attractor Landscape Analysis 11

2. Case Study 2 – Reprogramming p53-mediated Apoptosis in MCF-7 Breast Cancer Cell Lines 12

3. Case Study 3 – Investigating the Evolution of Cell Fate Landscape during Colorectal Tumorigenesis 16

C. Comparison of Features - ATLANTIS vs. Other Tools 19

D. Performance Analysis of ATLANTIS 20

E. Step by Step 24

F. Availability 27

G. Video Tutorials 28

H. Worked Examples 30

1. Decoding Yeast Cell Cycle Progression using Attractor Landscape Analysis 30

2. Investigating the Evolution of Cell Fate Landscape during Colorectal Tumorigenesis 32

3. Demonstrating the use of 'Trajectory Mapping' in Yeast Cell Cycle Progression Network . 34

4. Demonstrating the use of 'Fixed Nodes States' feature in 201-node network for Colorectal Tumorigenesis 37

5. Demonstrating the use of 'Custom States' feature in PA of Yeast Cell Cycle Progression Network 39

Supplementary References 41

A. Supplementary Methods

1. Data Preparation for use with ATLANTIS

ATLANTIS takes three different files as input, including (i) network information file, (ii) network modification file and (iii) cell fate determination logic file. These files contain description of biomolecular networks and may be formatted as flat text files ('.txt') or comma separated files ('.csv'). Flat text files ('.txt') can be generated using Microsoft Notepad®, Notepad++[1], Sublime Text[2] or any suitable text editor. Comma separated files ('.csv') can be created using a spreadsheet software such as Microsoft Excel®, LibreOffice® and Apple Numbers®. Below, we provide the details of each file processed by ATLANTIS.

Network Inputs for Logic Weights Based DA: This file contains two types of data, (i) node interaction weights matrix and (ii) a vector of node basal values indicating background expression of each node. Figure S1 (a, b) shows the contents of a sample tab-delimited text and a spreadsheet data file for generating network. The interaction weight matrix contains node interaction strengths (termed *interaction weights*) augmented with their names within the file.

Network Inputs for Rules Based DA: In case of rules-based DA, two inputs are required. These include: (i) a node list file containing names of all the nodes present in the network, and (ii) the path of a folder containing node state update logic files for each node in the node list file. Each node state update logic file contains information on the target node, regulatory nodes and the rules defining target node activity.

Network Modification File: Biomolecular networks can be perturbed by mutating biomolecules within the network. Information for mutating the nodes and their interactions is provided as a separate file. These mutations can be introduced into the network in two different ways. Firstly, the network can be modified by altering node states (either from 0 to 1 or from 1 to 0). Secondly, modifications can also be made by deleting the interaction link between two nodes. To modify a user-defined network, for use in ATLANTIS, a simple text file (Figure S1c) or a comma separated file (Figure S1d) is required. These files can be used to mutate a node by providing node names and their altered (i.e. mutated) states (Figure S1c, d) or by removing the interaction between two nodes through providing names of interacting nodes (e.g. A and B) in the following format 'A>B 2' (Figure S1c, d). ATLANTIS identifies link deletion between two nodes by the label '2' which should be followed by the two node names.

Cell Fate Determination Logic File: Cell fates are specific network states constituted by combinations of node states. Biologically relevant network states are associated to cell fates by providing cell fate determination logic in the form of a spreadsheet ('.csv') file. In this file, the user can provide node state combinations pertaining to each cell fate. For instance, the node states of P53, P21, Cyclin-E and Caspase corresponding to cell fate "cellular proliferation" are 0, 0, 1 and 0 respectively (Figure S1e). Therefore, the network state arising out of this node state combination is 0010 and the associated cell

fate is cellular proliferation. An example spreadsheet for specifying the cell fate logic is provided in Figure S1e.

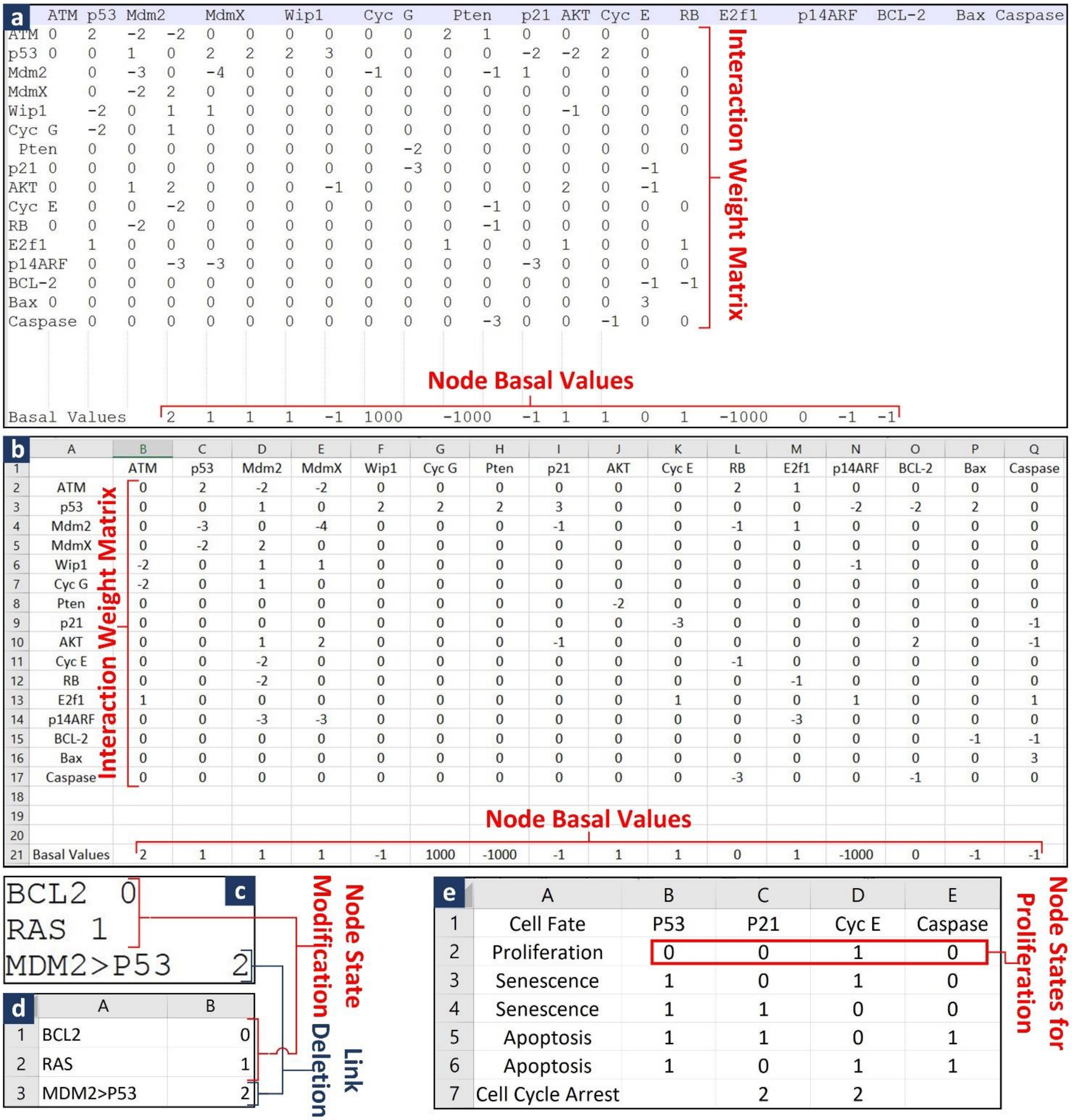


```
     ATM p53 Mdm2    MdmX    Wip1    Cyc G    Pten    p21 AKT Cyc E    RB  E2f1    p14ARF  BCL-2   Bax Caspase
ATM 0    2    -2  -2  0   0   0   0   0   0   2   1   0   0   0   0
p53 0    0    1   0   2   2   2   3   0   0   0   0   -2  -2  2   0
Mdm2     0    -3  0   -4  0   0   0   -1  0   0   -1  1   0   0   0   0
MdmX     0    -2  2   0   0   0   0   0   0   0   0   0   0   0   0   0
Wip1     -2   0   1   1   0   0   0   0   0   0   0   0   -1  0   0   0
Cyc G    -2   0   1   0   0   0   0   0   0   0   0   0   0   0   0   0
 Pten    0    0   0   0   0   0   0   0   -2  0   0   0   0   0   0   0
p21 0    0    0   0   0   0   0   0   0   -3  0   0   0   0   0   -1
AKT 0    0    1   2   0   0   0   -1  0   0   0   0   0   2   0   -1
Cyc E    0    0   -2  0   0   0   0   0   0   0   -1  0   0   0   0   0
RB   0   0    -2  0   0   0   0   0   0   0   0   -1  0   0   0   0
E2f1     1    0   0   0   0   0   0   0   0   1   0   0   1   0   0   1
p14ARF   0    0   -3  -3  0   0   0   0   0   0   0   -3  0   0   0   0
BCL-2    0    0   0   0   0   0   0   0   0   0   0   0   0   0   -1  -1
Bax 0    0    0   0   0   0   0   0   0   0   0   0   0   0   0   3
Caspase  0    0   0   0   0   0   0   0   0   0   -3  0   0   -1  0   0

Basal Values    2    1   1   1   -1  1000    -1000   -1  1   1   0   1   -1000   0   -1  -1
```

	A	B	C	D	E	F	G	H	I	J	K	L	M	N	O	P	Q
1		ATM	p53	Mdm2	MdmX	Wip1	Cyc G	Pten	p21	AKT	Cyc E	RB	E2f1	p14ARF	BCL-2	Bax	Caspase
2	ATM	0	2	-2	-2	0	0	0	0	0	0	2	1	0	0	0	0
3	p53	0	0	1	0	2	2	2	3	0	0	0	0	-2	-2	2	0
4	Mdm2	0	-3	0	-4	0	0	0	-1	0	0	-1	1	0	0	0	0
5	MdmX	0	-2	2	0	0	0	0	0	0	0	0	0	0	0	0	0
6	Wip1	-2	0	1	1	0	0	0	0	0	0	0	0	-1	0	0	0
7	Cyc G	-2	0	1	0	0	0	0	0	0	0	0	0	0	0	0	0
8	Pten	0	0	0	0	0	0	0	0	-2	0	0	0	0	0	0	0
9	p21	0	0	0	0	0	0	0	0	0	-3	0	0	0	0	0	-1
10	AKT	0	0	1	2	0	0	0	-1	0	0	0	0	0	2	0	-1
11	Cyc E	0	0	-2	0	0	0	0	0	0	0	-1	0	0	0	0	0
12	RB	0	0	-2	0	0	0	0	0	0	0	0	-1	0	0	0	0
13	E2f1	1	0	0	0	0	0	0	0	0	1	0	0	1	0	0	1
14	p14ARF	0	0	-3	-3	0	0	0	0	0	0	0	-3	0	0	0	0
15	BCL-2	0	0	0	0	0	0	0	0	0	0	0	0	0	0	-1	-1
16	Bax	0	0	0	0	0	0	0	0	0	0	0	0	0	0	0	3
17	Caspase	0	0	0	0	0	0	0	0	0	0	-3	0	0	-1	0	0
18																	
19																	
20																	
21	Basal Values	2	1	1	1	-1	1000	-1000	-1	1	1	0	1	-1000	0	-1	-1

```
BCL2  0
RAS  1
MDM2>P53    2
```

	A	B
1	BCL2	0
2	RAS	1
3	MDM2>P53	2

	A	B	C	D	E
1	Cell Fate	P53	P21	Cyc E	Caspase
2	Proliferation	0	0	1	0
3	Senescence	1	0	1	0
4	Senescence	1	1	0	0
5	Apoptosis	1	1	0	1
6	Apoptosis	1	0	1	1
7	Cell Cycle Arrest		2	2	

Supplementary Figure S1. Network Information and Modification Files ('.txt' and '.csv'). (a) Flat text file containing interaction weight and node basal values. To incorporate MCF-7 characteristic mutations into the p53 signaling network[3], Cyc G node is overexpressed, while Pten and p14ARF nodes are knocked down by setting the basal expression to 1000 and -1000, respectively, (b) Spreadsheet containing interaction weight matrix and node basal values, (c) Flat text file containing node state modification and link deletion, (d)

Spreadsheet containing node state modification and link deletion, (e) Cell fate determination logic file, where ‘0’, ‘1’ and ‘2’ represent ‘on’, ‘off’ and ‘oscillatory’ (continuously flipping) states, respectively.

2. Boolean Modelling of Biomolecular Networks

Boolean models for gene regulatory networks as pioneered by Stuart A. Kauffman in 1969 postulated that genes could be prototyped as switches acting in concert, towards a specific cell fate[4,5]. The approach formulated biomolecular network topology as a set of *nodes*, conjoined by a set of *links*, serving as a simplistic yet scalable approach towards capturing network dynamics. For modelling purposes, Boolean networks with ‘n’ nodes (where each node could represent a gene, a protein or any biomolecule) can be represented by a binary node vector, $N = [x_1, x_2, \dots, x_n]$. Each node in the vector, N, can take on two possible values, either 0 (denoting the inactive, unexpressed state of the biomolecule) or 1 (indicating active, expressed state of the biomolecule). Each network can, therefore, have a total of 2^n states, where ‘n’ is the total number of nodes (or biomolecules) in the network[6,7]. Figure S2 portrays a three-node network, having a total of $2^3 = 8$ states. These states are maintained in a *state space* matrix, $S = [s_{ij}]_{2^n \times n}$. The 3-node network shown in Figure S2 has its state space matrix represented by an 8×3 matrix, with each row representing a unique network and node state. Network evolution can be visualized using state transition plots. Each network state can, therefore, be termed as a biomolecular activity profile of the network at any given time instant[7].

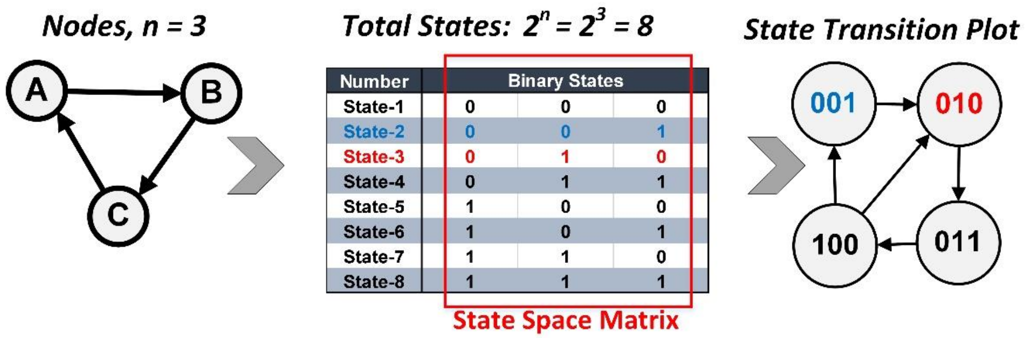

Supplementary Figure S2. A Simplistic Node-Link Graph and its State Space. A three-node network has its *state space* comprising of eight states. The system can evolve from one network state to another, the dynamics of which can be visualized by a state transition plot (State-2, 001, (shown in blue) to State-3, 010, (shown in red)).

3. State Transitions and Network Dynamics

After computing the complete state space of any given network, we can define the constituent node state transition rules. Transition between two states depends upon a general transition function, f, that governs the change in state (or value) of a single node, x_i. Therefore, to compute the change in state of a node, the transition function, f, considers all the parent nodes (activating or suppressing) of

the target node, x_i, at any time instant, t. Given, x_i has k parents, then the transition function from t to $t+1$ can be given by the equation as follows:

$$x_i(t+1) = f\big(x_1(t), x_2(t), \dots, x_j(t) \dots, x_k(t)\big) \ , k \leq \ n$$

Given that the transition function makes use of k parent state values for updating the state of a node, at the next time step, a generic mathematical formulation for Boolean modelling has been derived[6,7]. These formulations for Boolean networks envisage two modelling paradigms[6], (i) deterministic models, that represent a closed system with no external noise (or external input/outputs) and (ii) probabilistic models, representing open (noisy) systems hosting external noise (or inputs/outputs). Cellular and extracellular noise can also be termed as system perturbations[6,7].

Using ATLANTIS, users can perform deterministic as well as probabilistic modelling for cell fate discovery and reprogramming. Below, we detail on the node state transition mechanisms employed by ATLANTIS for deterministic analysis (DA) and probabilistic analysis (PA) respectively.

4. Deterministic Analysis of Boolean Networks

In DA, a biomolecular network is represented as a closed system, assuming no external noise or extracellular signaling perturbations. Apart from the general Boolean model dynamics (discussed earlier), DA pipeline makes use of a node basal value vector, $B = [b_1, b_2, \dots, b_n]$, provided by the user. This vector contains biomolecular expression values indicating basal activity of each node. Basal activity values are node expressions without any input or bias. The interaction weight matrix, $I_w = [w_{ij}]_{n \times n}$, represents the nature and effective mass of interaction between two participating nodes. The node-interaction model, thus formulated, can be simulated via a node state transition function. To update node states using DA, a transition function integrates each model component including basal values, interaction weights and node states[3] as given by the piece-wise equation below:

$$x_i(t+1) = \begin{cases} 1 & if \ \sum_j \big(w_{ji}x_j(t)\big) + b_i > 0 \\ 0 & else \ \ if \ \sum_j \big(w_{ji}x_j(t)\big) + b_i < 0 \\ x_i & else \ \ if \ \sum_j \big(w_{ji}x_j(t)\big) + b_i = 0 \end{cases}$$

The equation above defines node (x_i) state transition from time step t to $t+1$. The summation, $\sum_j \big(w_{ji}x_j(t)\big) + b_i$, gives the cumulative weighted interactions and the basal value. The result from the expression is thresholded using a *sign* (*Signum* function) or a *sigmoid* function (Figure S3). ATLANTIS employs a *sign* function in this case (Figure S3) which is defined as follows: [5]

$$x_i(t+1) = f(\sum_j \big(w_{ji}x_j(t)\big) + b_i)$$

where, f is *Signum* (or *Sign*) function defined over any input variable, z , as follows: [5]

$$f(z) = \begin{cases} 1 & if\ z > 0 \\ 0 & if\ z = 0 \\ -1 & if\ z < 0 \end{cases}$$

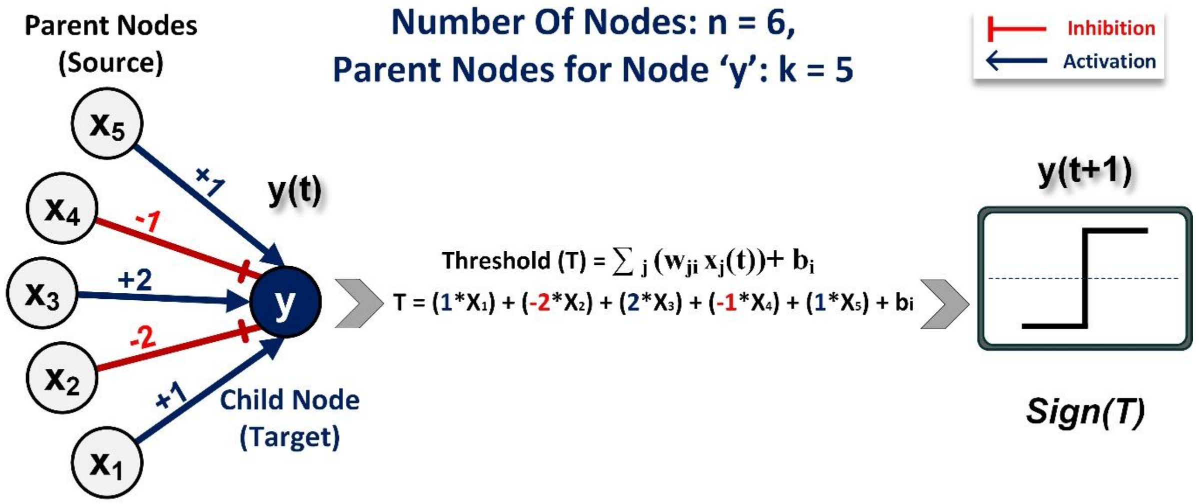

Supplementary Figure S3. Node Update for Deterministic Analysis in ATLANTIS. The example network has 1 target node, y, which has 5 parent nodes ($k = 5$) regulating it. Three of these parents are activating the node, y, while the remaining two are inhibiting its activity. Interaction weights indicate the magnitude of regulation of the target node by its parents. For deterministically updating the state of the node, y, from time, t to $t + 1$, a threshold value, T, is computed followed by its use by a *sign* function that sets up updated node state.

Figure S4 outlines the workflow of the DA pipeline implemented in ATLANTIS. The DA pipeline starts with a set of initial network states that can be generated by (1) exhaustive sampling (ES) of the state-space, (2) random sampling (RS) of the state-space or (3) inputting a custom state-space (CS) file. ES generates all (2^n) possible states for onward analysis. RS selects a user-specified number of states from the complete state-space, thereby making the analysis of larger networks feasible. Using the CS option, ATLANTIS users can selectively incorporate specific network states for further analysis. To achieve steady state, network states and transition functions are used. Once the system attains steady-state, the frequently recurring states (or *attractors*) are identified for attractor landscape plotting (discussed later).

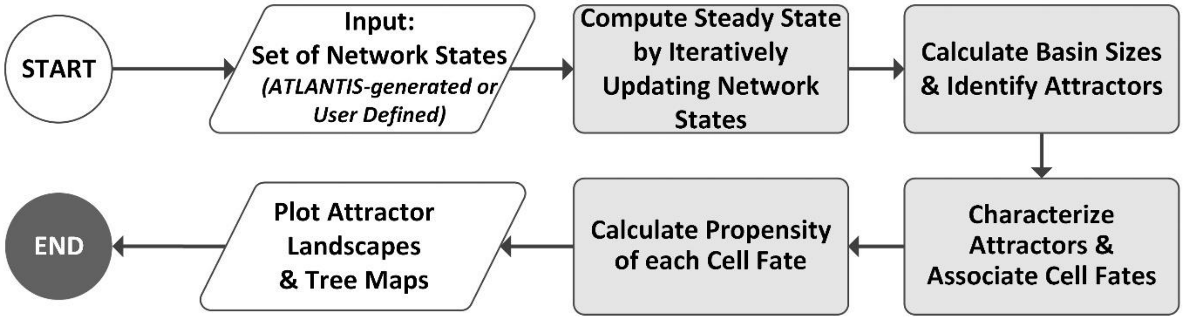

Supplementary Figure S4. Deterministic Analysis Pipeline Implemented by ATLANTIS.

Rules Based Deterministic Analysis

To assist in cell fate discovery process, ATLANTIS users can incorporate specific node update rules into the DA pipeline. Node update rules are employed during network update and stored in the form of truth tables. State transition rules are constructed from the exhaustive state space and only the biologically plausible node state transitions are employed onwards. Therefore, each node state gets updated by a rules look-up from the rules table. An example of rules-based DA is given below in Figure S5. The child node 'C' with parents 'A' and 'B' is updated to state '0' by applying the rule '0 1 0' (in red box).

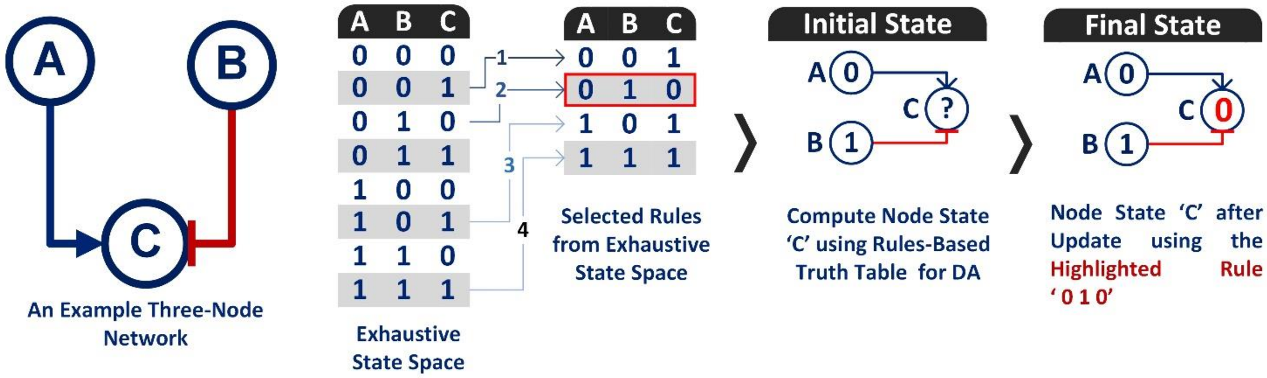

Supplementary Figure S5. Rules-Based Deterministic Analysis Methodology. A three-node network, with two parent nodes, 'A' and 'B' and one child node, 'C' is considered for rules-based DA. The state of node 'C' ('0') is computed by using rules-based logic defined (red box) from within the exhaustive state space.

5. Probabilistic Analysis of Boolean Networks

The probabilistic analysis (PA) pipeline of ATLANTIS (Figure S6) works off a biomolecular network input with nodes and interaction weights. As in the case of DA, PA can also employ exhaustive, randomly sampled or user-defined custom state-space for onwards analysis. However, unlike DA, the transitions between network states follows a *Markovian* model in PA. Network state at the next time step, $t+1$, is determined only by its state at the current time step, t, with no dependence on earlier time steps. As a result, the state transition probability from t to $t+1$ is a product of individual node transition probabilities from t to $t+1$[5, 6]:

$$\mathrm{T}\{x_i(t+1), x_2(t+1), \dots x_n(t+1) | x_1(t), x_2(t), \dots x_n(t)\} = \prod_{i=1}^{n} T\,\{x_i(t+1) | x_1(t), x_2(t), \dots x_n(t)\}$$

To integrate the effect of background basal-level activity of nodes, ($B = [b_1, b_2, \dots, b_n]$), in absence of any input, the cumulative inputs to each node are added with its basal expression. Intrinsic and extrinsic noise is incorporated with use of the noise parameter[5], μ. An additional parameter c is used to quantify self-degradation of a node[5]. Given that the nodes lie within a dynamic system, the output from a single node is computed using the cumulative effect of its interaction with the parent

nodes, noise, basal values and self-degradation constant. The input can be either positive, negative or zero, according to which the node transition probability is updated. The transition matrix T for each node[6] is defined as follows:

$$T\{x_i(t+1)=1|x_1(t),x_2(t),\dots x_n(t)\}=\frac{1}{2}+\frac{1}{2}\tanh\left[\mu\left(\sum_{j=1}^{n}\left(w_{ji}x_j(t)\right)+b_i\right)\right]\quad if\ \sum_{j=1}^{n}\left(w_{ji}x_j(t)\right)+b_i>0$$

$$T\{x_i(t+1)=0|x_1(t),x_2(t),\dots x_n(t)\}=\frac{1}{2}-\frac{1}{2}\tanh\left[\mu\left(\sum_{j=1}^{n}\left(w_{ji}x_j(t)\right)+b_i\right)\right]\quad if\ \sum_{j=1}^{n}\left(w_{ji}x_j(t)\right)+b_i<0$$

$$T\{x_i(t+1)=x_i(t)|x_1(t),x_2(t),\dots x_n(t)\}=1-c\qquad if\ \sum_{j=1}^{n}\left(w_{ji}x_j(t)\right)+b_i=0$$

If the input $\sum_{j=1}^{n}\left(w_{ji}S_j(t)\right)+b_i$ received by a node i is positive (>0), the probability of transitioning to an ON state is high and its probability of transitioning to an OFF state is low. Conversely, if the input $\sum_{j=1}^{n}\left(w_{ji}S_j(t)\right)+b_i$ is negative (<0), the node will transition to an OFF state with a high probability but its transition to an ON state will have a low probability. The last condition defines the probability of a node staying in the same state at the next time step, as there is essentially no change in the input coming into the node. In such a case, the transition probability is dependent on the self-degradation of the node with a value equal to $1 - c$.[6]

Following computation of network transition probabilities, it is important to see how the states evolve to reach a *steady-state*, such that there is no further change in their probabilities. This requires the use of kinetic master equations to update state probabilities over time[6] and is given below:

$$\frac{dP_i}{dt}=-\sum_{j}\mathrm{T}_{ij}P_i+\sum_{j}\mathrm{T}_{ji}P_j$$

The state transition probabilities change over time due to the effect of neighboring network states according to transitions in both directions from i to j, and j to i. T_{ij} is the probability of a network state transitioning from a network state i to j, and the *vice versa* for T_{ji}. i and j iterate over all the states (i.e. from 1 to 2^n). The steady-state probabilities are stored in variables, P_i and P_j, wherein all states are initially assigned equal probabilities[6] ($P_i=\frac{1}{2^n}$). After that, each P_i is dynamically updated until a steady state, i.e. $P_i(t+1)$ equals $P_i(t)$ to good approximation. For tractable computation in large networks, the state-space can also be pruned using one-step DA (termed Heuristic PA). Finally, a potential energy (*PE*) value is assigned to each state to represent its stability, which is then used to construct PE landscapes for visualizing PA results.

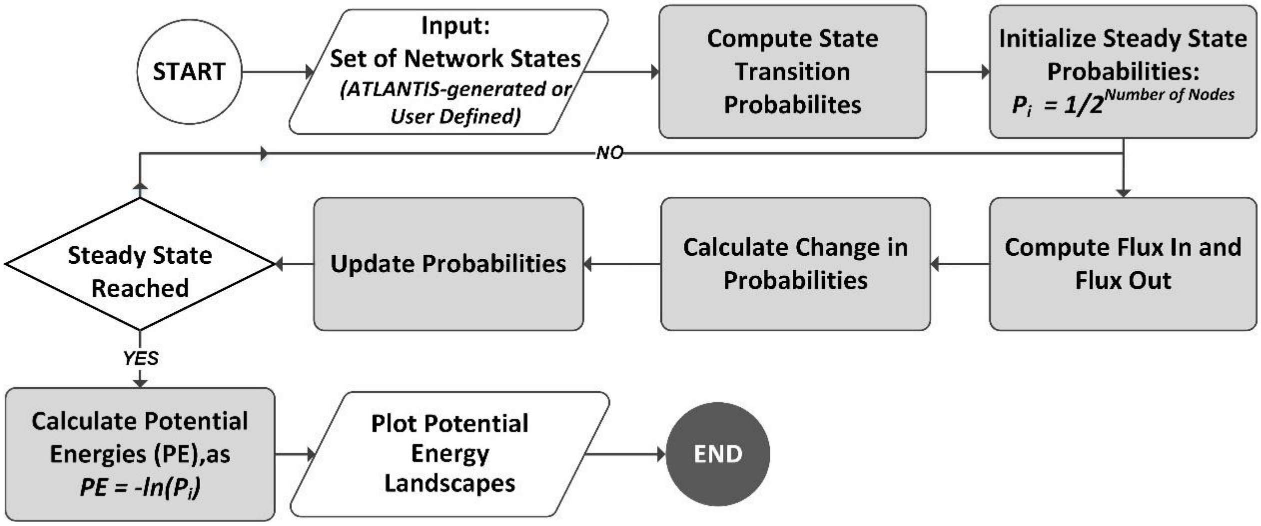

Supplementary Figure S6. Probabilistic Analysis Pipeline Implemented in ATLANTIS.

6. Plotting Attractor and Cell Fate Landscape

After the system attains its steady state using DA or PA, the most frequently occurring attractor states are extracted. In DA pipeline, attractor states are identified by their basin sizes (frequency of occurrence of each network state). In case of PA, each network state's associated probability (likelihood of its emergence) defines the attractor states. These attractor states are then plotted and are termed as *attractor landscapes*. For plotting attractor landscapes, the high dimensional state space is projected onto a two-dimensional coordinate space using two approaches. These include Naïve mapping and *Sammon* mapping[8]. Naïve mapping maps the state-space onto a Cartesian plane without considering the spatial distance between different steady-states. Sammon mapping, on the other hand, clusters the related network states together before their projection onto a Cartesian plane.

In case of PA, potential energies (PE) are plotted as PE landscapes. These PEs are derived from steady-state probabilities as follows:

$$PE_i = -\ln(P_i)$$

Probability landscape provides a visualization of steady-state probabilities, whereas PE landscape provides an energetics perspective of the probabilities. Scaling by natural logarithm in PEs amplifies the distance between the attractor and low propensity states thereby imparting more clarity to PE landscapes. Additionally, ATLANTIS also provides for constructing cell fate landscapes (CFL). After DA, a treemap is constructed using basin sizes of attractors. Each attractor is then correlated to various cellular fates (e.g. proliferative or apoptotic) based on user-defined cell fate logic.

7. HCT-116 Characteristic Mutations

In case study 4, ATLANTIS was used to discover cell fates including normal proliferation, abnormal proliferation, metastasis, and quiescence adopted by HCT-116 cells in control and post-inhibitor treatment. For this purpose, an HCT-116 representative network was constructed by adding cell line characteristic mutations (Table S1) to the previously reported human CRC signaling network[9]. The cell line tailored network was deterministically analyzed. The resulting attractors were characterized and associated with the aforementioned cell fates.

**Supplementary Table S1| HCT-116 Characteristic Mutations**			
**Cancer Genes**	**Protein sequence**	**Pathogenicity**	**Mutation Value**
RAS	p.G13D	Pathogenic (score 0.98)	1
P14arf	p.R24fs*20	None (score 0.00)	
β-Catenin	p.S45del	None (score 0.00)	
PI3K	p.H1047R	Pathogenic (score 0.96)	1

Supplementary Table S1. HCT-116 Characteristic Mutations Adapted from COSMIC Database[10].

B. Supplementary Results

Functionality validation of ATLANTIS was undertaken by constructing four case studies including (1) Yeast cell cycle analysis[11], (2) p53 network analysis[3], (3) analysis of tumorigenesis in human colon[9], and (4) drug-target interaction in HCT-116 cell lines. Results for probabilistic and deterministic analyses, basin ratios and attractor landscapes were compared with the results obtained from published literature for each case study. The supplementary results obtained from these analyses are described in the sub-sections below, along with their comparison with published data.

1. Case Study 1 – Decoding Yeast Cell Cycle Progression using Attractor Landscape Analysis

The first case study was performed by reconstructing the study "*Yeast Cell Cycle Network: The Funneled Energy Landscape*", reported earlier by Han *et al*[11]. The cell cycle regulatory network had 11 nodes and 34 edges (Figure S7a). ATLANTIS was used to construct the attractor landscape of the cell cycle network (Figure S7b) and basin sizes were computed using deterministic and probabilistic analyses (DA and PA). Table S2 shows the high propensity network states constituting a state trajectory beginning at "Start signal" (state: 1093) and terminating at "Stationary G1 phase" (state: 69). ATLANTIS successfully reproduced the "Order in Trajectory" as reported by Han *et al*. More so, basin size of "Stationary G1 phase" reported by ATLANTIS was comparable with the value obtained from BoolNet[12]. Note that the basin sizes of off-pathway traps[11] (states: 385, 581, 1, 5, 65, 517) were also consistently reported by both ATLANTIS and BoolNet.

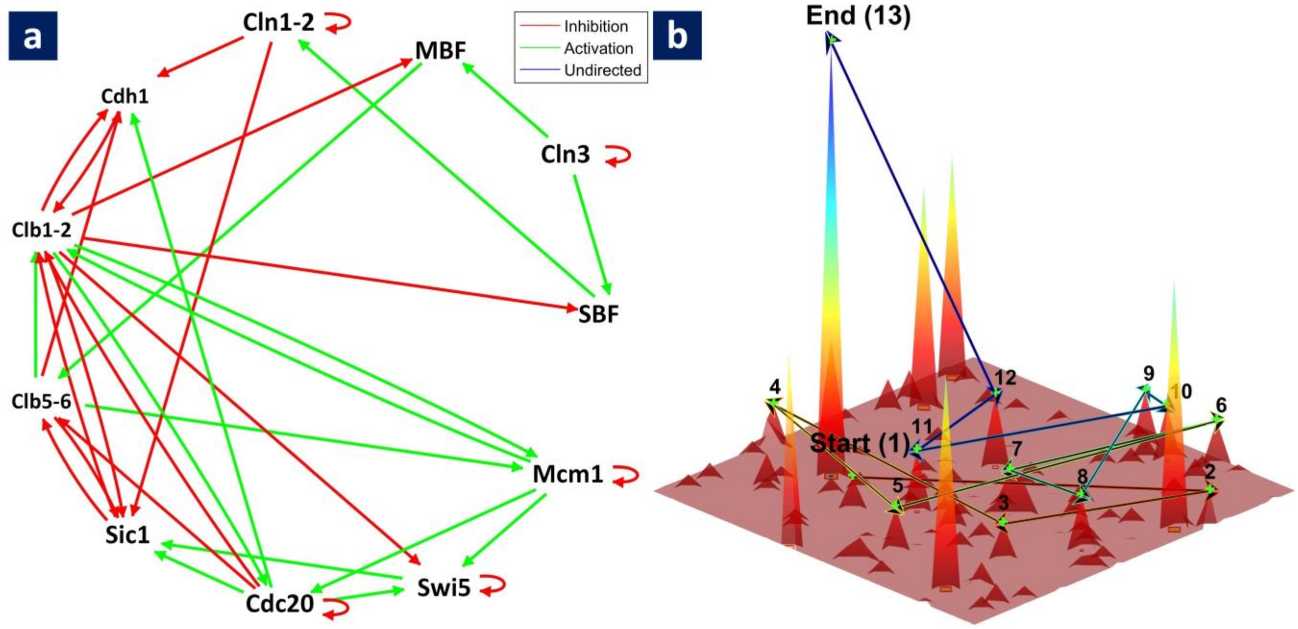

Supplementary Figure S7. Decoding Yeast Cell Cycle Progression. (a) Han *et al.*'s yeast cell cycle network generated using ATLANTIS, (b) Attractor landscape plot of recovered cell fates from yeast cell cycle network by ATLANTIS, showing the complete state transition trajectory from the 'start signal' to 'stationary G1' phase.

Supplementary Table S2 | Comparison of results from Yeast cell cycle network analyses																	
Network States											State #	*Han et al*		*ATLANTIS*			*BoolNet*
cln3	MBF	SBF	Cln1,2	Cdh1	Swi5	Cdc20	Clb5,6	Sic1	Clb1,2	Mcm1		Description	Order in Trajectory	Order in Trajectory	Steady State Probabilities	Basin Size	Basin Size
0	0	0	0	1	0	0	0	1	0	0	69	Stationary G1 phase	13	13	0.539	0.861	0.857
0	0	0	0	1	1	0	0	1	0	0	101	Excited G1 state	12	12	0.004	0	0
0	0	0	0	1	1	1	0	1	0	0	117	M phase	11	11	0.003	0	0
0	0	0	0	0	1	1	0	1	0	1	54		10	10	0.002	0	0
0	0	0	0	0	1	1	0	1	1	1	56		9	9	0.002	0	0
0	0	0	0	0	1	1	0	0	1	1	52		8	8	0.002	0	0
0	0	0	1	0	0	1	1	0	1	1	156		7	7	0.001	0	0
0	1	1	1	0	0	0	1	0	1	1	908	G2 phase	6	6	0.001	0	0
0	1	1	1	0	0	0	1	0	0	0	905	S phase	5	5	0.001	0	0
0	1	1	1	0	0	0	0	0	0	0	897	Excited G1 state	4	4	0.001	0	0
0	1	1	1	1	0	0	0	1	0	0	965		3	3	0.001	0	0
0	1	1	0	1	0	0	0	1	0	0	837		2	2	0.001	0	0
1	0	0	0	1	0	0	0	1	0	0	1093	Start signal	1	1	0.001	0	0
0	0	1	1	0	0	0	0	0	0	0	385	-	-	-	0.115	0.074	0.076
0	1	0	0	1	0	0	0	1	0	0	581	-	-	-	0.083	0.053	0.065
0	0	0	0	0	0	0	0	0	0	0	1	-	-	-	0.083	0.003	0.003
0	0	0	0	0	0	0	0	1	0	0	5	-	-	-	0.078	0.004	0.004
0	0	0	0	1	0	0	0	0	0	0	65	-	-	-	0.058	0.000	0.000
0	1	0	0	0	0	0	0	1	0	0	517	-	-	-	0.014	0.003	0.003

Supplementary Table S2. Comparison of Results from Yeast Cell Cycle Network Analyses. Results from state transition trajectory analysis computed by Han *et al*, ATLANTIS and BoolNet are enumerated for comparison after rounding off to three significant figures. The highest steady state probability (0.539) was observed for "Stationary G1 phase" state marking the end of the state transition trajectory. The state also had the highest basin size (0.861) which was consistently reported by both ATLANTIS and BoolNet. Moreover, the order of state transitions (1 to 13) within the trajectory after a "Start Signal", tallied with the results from Han *et al*.

2. Case Study 2 – Reprogramming p53-mediated Apoptosis in MCF-7 Breast Cancer Cell Lines

The second case study is built on "*Attractor landscape analysis reveals feedback loops in the p53 network that control the cellular response to DNA damage*" by Choi *et al*[3]. The P53 regulatory network (Figure S8) comprised of 16 nodes and 50 edges. ATLANTIS was used to construct the cell fate landscapes (CFL) (Figure S9) and compute basin sizes (Table S3) using DA. All possible combinations of Etoposide, Nutlin and WIP1 KD were tested and basin sizes as well as cell fates for each combination obtained from ATLANTIS and BoolNet were found to be in agreement. Next, to compare attractor landscapes, PA was performed to reconstruct the landscape reported by Choi *et al* for the combinatorial effect of Etoposide, Nutlin, and WIP1 KD. ATLANTIS accurately computed the landscape (Figure S10a) and identified *three* point attractors for cell senescence and 2 point attractors for cell death landscape as reported by Choi *et al* (Figure S10b).

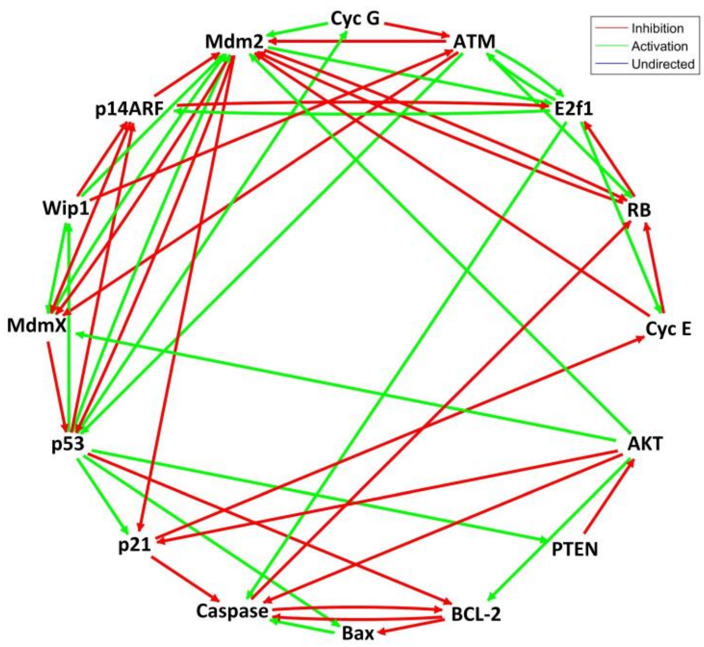

Supplementary Figure S8. P53-mediated Apoptotic Network for MCF-7 Cells Comprising of 16 Nodes and 50 Edges.

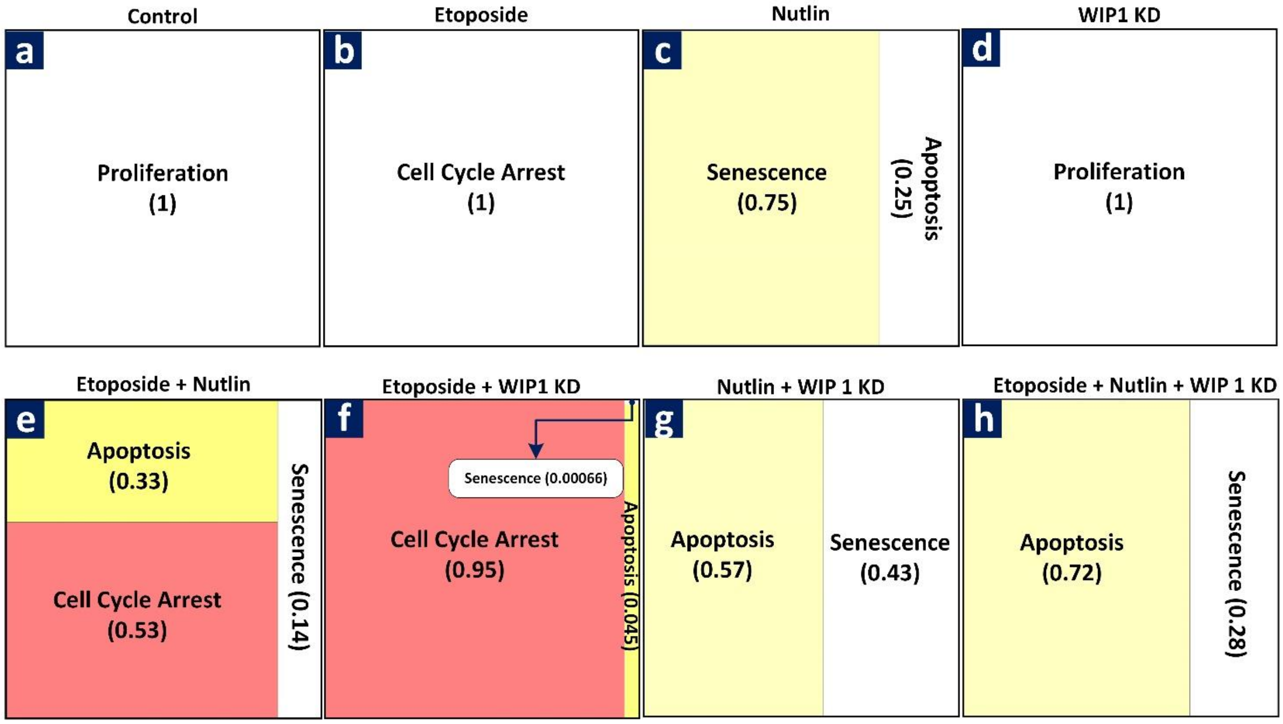

Supplementary Figure S9. MCF7 Cell Fate Propensity Landscape after Treatment with Etoposide, Nutlin and CCT007093/WIP1 KD. (a) Control (no treatment), (b) Etoposide treatment, (c) Nutlin treatment, (d) WIP1 knock-down, (e) combined Etoposide and Nutlin treatment, (f) combined Etoposide and WIP1 knock-down, (g) combined Nutlin and WIP1 knock-down, and (h) combined Etoposide, Nutlin and WIP1 knock-down treatment.

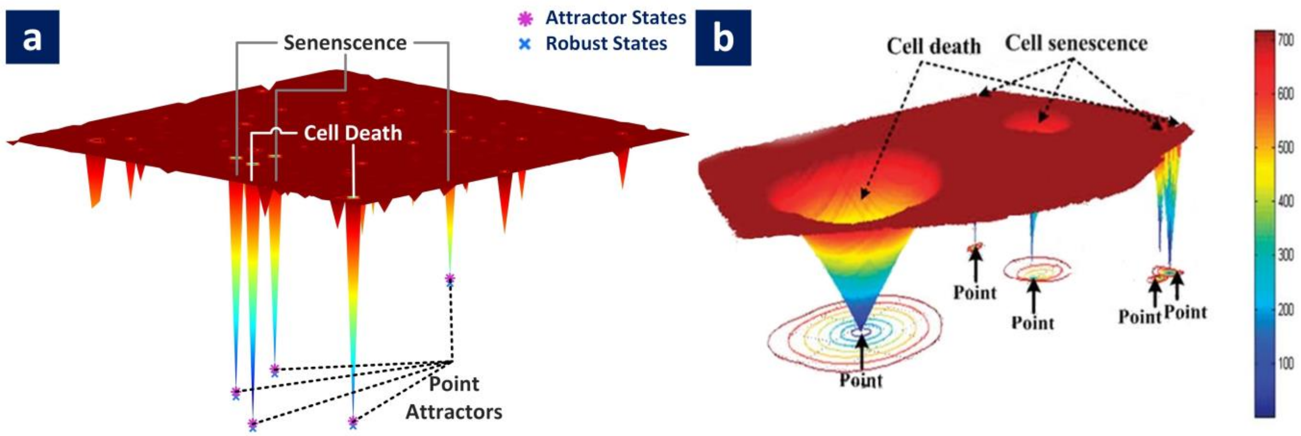

Supplementary Figure S10. Attractor Landscapes of MCF-7 Network after Combinatorial Treatment with Etoposide, Nutlin and CCT007093/WIP1 Knock-Down. (a) ATLANTIS-generated attractor landscape tallying each point attractor in (b) constructed using *Sammon* mapping, (b) attractor landscape as proposed in their work by Choi *et al*, containing 3 point attractors for cell senescence and 2 point attractors for cell death.

Supplementary Table S3 | Comparison of p53-mediated Apoptosis Network Analyses from Choi *et al*, ATLANTIS and BooNet																								
		Network States																			Choi *et al*	*ATLANTIS*		*BoolNet*
S/N	Condition	ATM	p53	Mdm2	MdmX	Wip1	Cyc G	Pten	p21	AKT	Cyc E	Rb	E2f1	p14ARF	BCL-2	Bax	Caspase	State #	Attractor Type	Cell Fate	Steady State Probabilities	Steady State Probabilities	Basin Size	Basin Size
1	Control	□	□	■	□	□	■	□	□	■	■	□	■	□	■	□	□	9429	Point	Proliferation	1.000	1.000	1.000	1.000
2	Etoposide	■	□	■	□	□	■	□	□	■	■	□	■	□	■	□	□	42197	Cyclic	Cell Cycle Arrest	0.143	0.300	1.000	1.000
		■	■	□	■	□	■	□	□	■	■	■	■	□	■	□	□	54517			0.143	0.180		
		□	□	■	■	□	■	□	□	■	□	□	■	□	■	□	□	13461			0.142	0.168		
		■	□	□	□	□	■	□	□	■	■	□	■	□	■	□	□	34005			0.143	0.116		
		■	■	□	■	■	■	□	■	■	■	■	■	□	■	□	□	56821			0.142	0.100		
		□	□	■	■	■	■	□	■	■	□	□	■	□	■	□	□	15765			0.141	0.098		
		□	■	■	■	■	■	□	■	■	□	■	■	□	■	□	□	32181			0.142	0.035		
3	Nutlin	□	■	■	□	■	■	□	□	■	■	□	■	□	■	□	□	27861	Point	Senescence	N/A	0.705	0.750	0.737
		□	■	■	□	■	■	□	□	■	■	□	■	□	□	■	■	27860		Apoptosis	N/A	0.148	0.124	0.145
		□	■	■	□	■	■	□	■	■	□	□	■	□	□	■	■	28052		Apoptosis	N/A	0.120	0.122	0.108
		□	■	■	□	■	■	□	■	■	□	□	■	□	■	□	□	28053		Senescence	N/A	0.016	0.002	0.005
		□	■	■	□	■	■	□	■	■	□	□	■	□	■	■	□	28055		Senescence	N/A	0.002	0.002	0.005
4	WIP1 KD	□	□	■	□	□	■	□	□	■	■	□	■	□	■	□	□	9429	Point	Proliferation	N/A	1.000	1.000	1.000
5	Nutlin + WIP1 KD	□	■	■	□	□	■	□	□	■	■	□	■	□	□	■	■	25812	Point	Apoptosis	N/A	0.461	0.452	0.342
		□	■	■	□	□	■	□	□	■	■	□	■	□	■	□	□	25813		Senescence	N/A	0.400	0.428	0.543
		□	■	■	□	□	■	□	■	■	□	□	■	□	□	■	■	26004		Apoptosis	N/A	0.123	0.116	0.105
		□	■	■	□	□	■	□	■	■	□	□	■	□	■	□	□	26005		Senescence	N/A	0.010	0.002	0.005
		□	■	■	□	□	■	□	■	■	□	□	■	□	■	■	□	26007		Senescence	N/A	0.002	0.002	0.005
6	Etoposide + Nutlin	■	■	□	■	■	■	□	■	■	■	■	■	□	■	□	□	56821	Cyclic	Cell Cycle Arrest	0.000	0.195	0.534	0.435
		□	■	■	■	■	■	□	■	■	□	■	■	□	■	□	□	32181			0.000	0.189		
		■	■	□	□	□	■	□	□	■	■	□	■	□	■	□	□	50389			0.148	0.076		
		□	□	■	■	■	■	□	■	■	□	□	■	□	■	□	□	15765			0.147	0.031		
		■	□	■	□	□	■	□	□	■	■	□	■	□	■	□	□	42197			0.148	0.022		
		□	□	■	■	□	■	□	□	■	□	□	■	□	■	□	□	13461			0.148	0.021		
		□	■	■	□	■	■	□	■	■	□	□	■	□	□	■	■	28052	Point	Apoptosis	0.079	0.199	0.206	0.198
		□	■	■	□	■	■	□	□	■	■	□	■	□	□	■	■	27860		Apoptosis	0.000	0.124	0.120	0.162
		□	■	■	□	■	■	□	□	■	■	□	■	□	■	□	□	27861		Senescence	0.000	0.110	0.115	0.152
		□	■	■	□	■	■	□	■	■	□	□	■	□	■	□	□	28053		Senescence	0.024	0.025	0.022	0.045
		□	■	■	□	■	■	□	■	■	□	□	■	□	■	■	□	28055		Senescence	0.007	0.003	0.003	0.008
7	Etoposide + WIP1 KD	■	■	□	■	□	■	□	■	■	□	■	■	□	■	□	□	54709	Cyclic	Cell cycle Arrest	0.161	0.217	0.954	0.964
		■	■	■	■	□	■	□	■	■	□	■	■	□	■	□	□	62901			0.161	0.186		
		■	■	□	■	□	■	□	□	■	■	■	■	□	■	□	□	54517			0.162	0.144		
		■	□	■	□	□	■	□	■	■	□	■	■	□	■	□	□	42421			0.161	0.140		
		■	□	□	□	□	■	□	□	■	□	■	■	□	■	□	□	33973			0.162	0.139		
		■	■	□	■	□	■	□	■	■	■	■	■	□	■	□	□	54773			0.162	0.122		
		■	■	■	□	□	■	□	■	■	□	□	■	□	□	■	■	58772	Point	Apoptosis	0.016	0.040	0.042	0.026
		■	■	■	□	□	■	□	□	■	■	□	■	□	□	■	■	58580		Apoptosis	0.007	0.004	0.003	0.008
		■	■	■	□	□	■	□	■	■	□	■	■	□	■	□	□	58805		Senescence	0.001	0.000	0.000	0.001
		■	■	■	□	□	■	□	□	■	■	□	■	□	■	□	□	58581		Senescence	0.000	0.000	0.000	0.000
		■	■	■	□	□	■	□	■	■	□	■	■	□	■	■	□	58807		Senescence	0.000	0.000	0.000	0.001
8	Etoposide + Nutlin + WIP1 KD	■	■	■	□	□	■	□	■	■	□	□	■	□	□	■	■	58772	Point	Apoptosis	0.000	0.538	0.538	0.476
		■	■	■	□	□	■	□	□	■	■	□	■	□	□	■	■	58580		Apoptosis	0.000	0.187	0.181	0.142
		■	■	■	□	□	■	□	■	■	□	■	■	□	■	□	□	58805		Senescence	0.000	0.175	0.180	0.267
		■	■	■	□	□	■	□	□	■	■	□	■	□	■	□	□	58581		Senescence	0.000	0.085	0.095	0.101
		■	■	■	□	□	■	□	■	■	□	■	■	□	■	■	□	58807		Senescence	0.000	0.009	0.006	0.014

Supplementary Table S3. Comparison of Results from p53-mediated Apoptosis Network Analyses. Steady-state probabilities of several cell fates were computed using probabilistic analysis in ATLANTIS and results were compared with those from Choi *et al*. Basin sizes for various cell fates were then computed using deterministic analysis in both ATLANTIS and BoolNet. The results from probabilistic as well as deterministic analyses compared well and ATLANTIS accurately classified cell fates including proliferation, cell cycle arrest, apoptosis and senescence.

3. Case Study 3 – Investigating the Evolution of Cell Fate Landscape during Colorectal Tumorigenesis

In this case study, we analyzed human signaling network constructed by Cho *et al* for evaluating colorectal tumorigenesis (CRC)[9]. The 201 nodes and 688 edges Boolean network is an integration of pertinent signaling pathways such as proliferation, metastasis, and apoptosis. ATLANTIS was employed to deterministically analyze the network. Sequential mutations were introduced into the network followed by construction of cell fates landscapes (Figure S11-S15). Basin sizes were used to compute cell fates which were then compared with Cho *et al* and resulting landscapes were found to be in agreement.

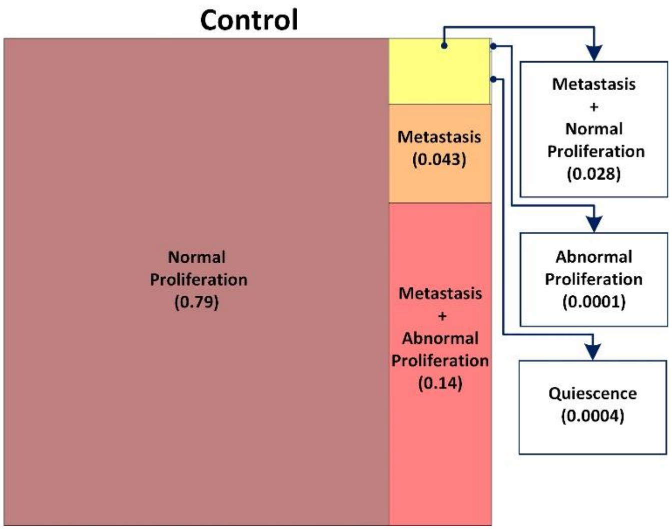

Supplementary Figure S11. Cell fate Landscapes of Colorectal Tumorigenesis Network in Control Condition. Reconstruction of cell fate landscape (treemap) constructed utilizing the network logic presented by Cho *et al* during control conditions.

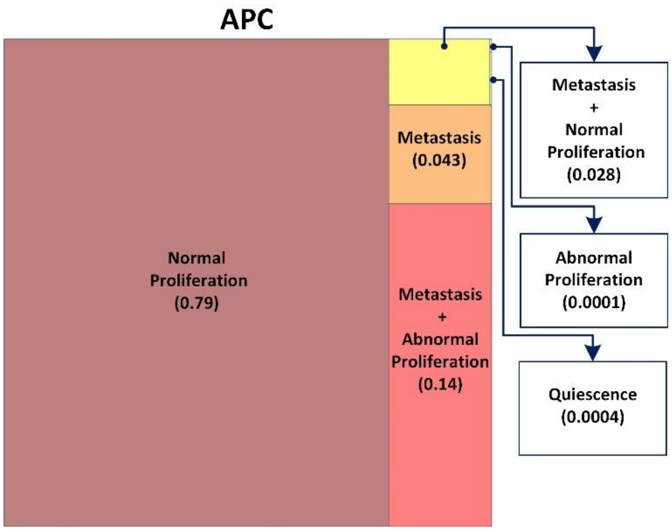

Supplementary Figure S12. Cell fate Landscapes of Colorectal Tumorigenesis Network after mutating APC. Reconstruction of cell fate landscape (treemap) constructed utilizing the network logic presented by Cho *et al* for mutated APC gene.

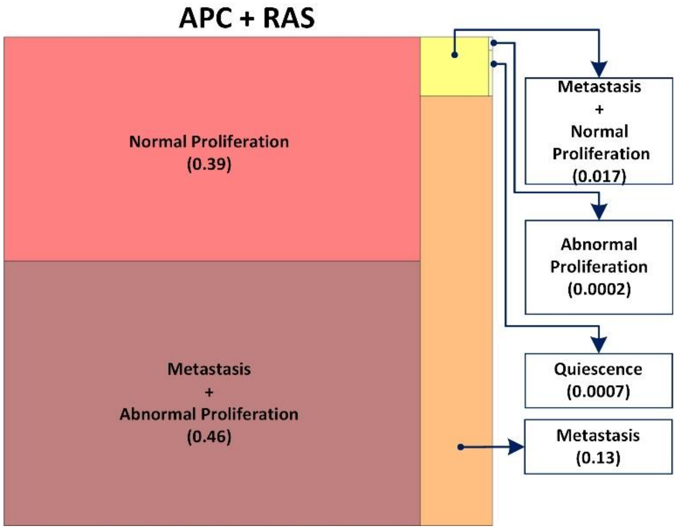

Supplementary Figure S13. Cell fate Landscapes of Colorectal Tumorigenesis Network after mutating APC and RAS. Reconstruction of cell fate landscape (treemap) constructed utilizing the network logic presented by Cho *et al* for mutated APC and RAS.

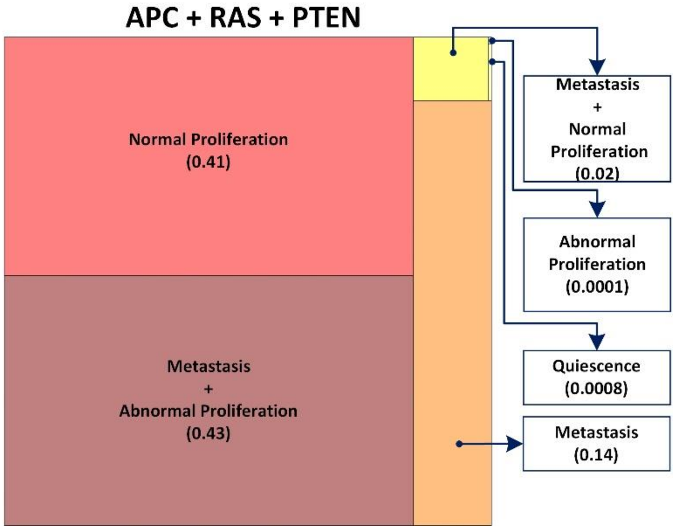

Supplementary Figure S14. Cell fate Landscapes of Colorectal Tumorigenesis Network after mutating APC, RAS and PTEN. Reconstruction of cell fate landscape (treemap) constructed utilizing the network logic presented by Cho *et al* for mutated APC, RAS and PTEN.

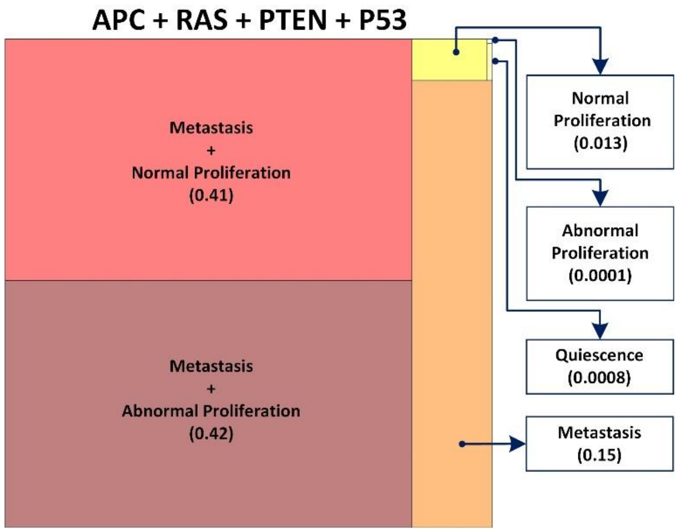

Supplementary Figure S15. Cell fate Landscapes of Colorectal Tumorigenesis Network after mutating APC, RAS, PTEN and P53. Reconstruction of cell fate landscape (treemap) constructed utilizing the network logic presented by Cho *et al* for mutated APC, RAS, PTEN and P53.

C. Comparison of Features - ATLANTIS vs. Other Tools

In this section, we have compared the salient features of ATLANTIS with contemporary Boolean network analysis tools aimed at computing attractor states. Below, a categorical list of features has been tabulated and the availability of these features is compared across each tool.

CATEGORIES	FEATURES	ATLANTIS	BoolNet	BooleSim	The Cell Collective	CellNetAnalyzer	GINsim	ViSiBooL	CABeRNET
Network State Space Modelling	Exhaustive State-space Generation	Green	Green	Orange	Orange	Green	Green	Orange	Green
	Custom State-space Generation	Green	Green	Green	Green	Orange	Green	Green	Green
	Random Subsets of State-space	Green	Green	Orange	Orange	Orange	Green	Orange	Orange
Types of Analyses	Logic Rules-based Determinstic Analysis (DA)	Green	Green	Green	Green	Green	Orange	Green	Green
	Interaction Weights-based DA	Green	Orange	Orange	Orange	Green	Green	Orange	Orange
	Probabilistic Analysis (PA)	Green	Green	Orange	Orange	Orange	Orange	Orange	Orange
Types of Perturbation	Node Knock-down	Green	Green	Green	Green	Green	Green	Green	Green
	Node Overexpression	Green	Green	Green	Green	Green	Green	Green	Green
	Link Deletion	Green	Orange	Orange	Orange	Green	Green	Orange	Orange
Attractor Landscape Analysis & Cell Fate Determination	Mapping Network States to Cell Fates	Green	Orange	Orange	Orange	Orange	Orange	Orange	Orange
	Computing Cell Fate Propensities	Green	Orange	Orange	Orange	Orange	Orange	Orange	Orange
	Cell Fate Landscapes	Green	Orange	Orange	Orange	Orange	Orange	Orange	Orange
	Basin Size Landscapes	Green	Orange	Orange	Orange	Orange	Orange	Orange	Orange
	Probability Landscapes	Green	Orange	Orange	Orange	Orange	Orange	Orange	Orange
	Potential Energy Landscapes	Green	Orange	Orange	Orange	Orange	Orange	Orange	Orange
Others	Graphical User Interfaces	Green	Orange	Green	Green	Green	Green	Green	Green
	Network Visualization	Green	Green	Green	Green	Green	Green	Green	Green
	Interactive Result Visualization	Green	Orange	Orange	Green	Orange	Green	Orange	Green
	Open Source	Green	Green	Green	Green	Orange	Green	Green	Orange

Supplementary Table S4. Comparison of Features between ATLANTIS with other Tools. ATLANTIS has been compared with BoolNet[12], BooleSim[13], The Cell Collective[14], CellNetAnalyzer[15], GINsim[16], ViSiBooL[17] and CABeRNET[18]. Features from Network State Space Modelling, Types of Analyses, Types of Perturbation, Attractor Landscape Analysis & Cell Fate Determination, and Others categories have been compared. Green and Orange boxes indicate presence and absence of specific features, respectively.

D.Performance Analysis of ATLANTIS

We have benchmarked the computational runtimes of case studies 1-4 towards apprising ATLANTIS users of the approximate time required to undertake such analyses. For this purpose, three hardware configurations were utilized, in light of typical computing infrastructures available to researchers. These configurations included a Laptop (Intel® Core i3-3120M @2.50GHz, 4GB RAM), a Desktop (Intel® Core i7-7700 @3.60GHz, 32GB RAM) and a Server (Dell® Power Edge R730, Dual Intel Xeon® E5-2620 @2.40GHz, 128GB RAM). The runtime benchmarking results obtained are tabulated in Supplementary Tables S5-S7.

Overall runtimes of case studies (from 1 to 4) have been summarized in Supplementary Table S5. For case study 1, deterministic and probabilistic analyses (DA and PA) were benchmarked to take between 2-7 seconds (see Supplementary Table S5). For case study 2, DA took between 4-12 seconds, while PA (with heuristically pruned state-space) took nearly 2 seconds. PA with exhaustive state-space took 206 seconds to run on Server configuration (see Supplementary Table S5). The limited memory sizes available in Laptop and Desktop configurations inhibited exhaustive network state-space analysis in case study 2. DA in case studies 3 and 4 were benchmarked to take between 1095-3515 seconds, depending on the hardware configuration employed (see Supplementary Table S5).

Overall Runtimes for Case Studies 1-4 using ATLANTIS		Computational Time (*in seconds*)		
		Laptop Configuration (CPU: 2.50 GHz, RAM: 4 GB)	Desktop Configuration (CPU: 3.60 GHz, RAM: 32 GB)	Server Configuration (CPU: 2.40 GHz, RAM: 128 GB)
Case Study 1	Deterministic Analysis	2	2	2
	Exhaustive Probabilisitc Analysis	7	4	6
Case Study 2	Deterministic Analysis	≤ 12	≤ 4	≤ 8
	*Heuristic Probabilisitc Analysis	2	2	2
	Exhaustive Probabilisitc Analysis	FTC	FTC	≤ 206
Case Study 3	Deterministic Analysis	≤ 3515	≤ 1410	≤ 2074
Case Study 4	Deterministic Analysis	≤ 2662	≤ 1095	≤ 1598

*Heuristically pruned state-space was employed in perform probablistic analysis FTC: Failed to compute

Note: For case studies 1 and 2, the "Simulation Timesteps" were set at 3000 and 17000, respectively.

From 3 repeated runs, only the largest runtimes have been reported for ATLANTIS

Supplementary Table S5. Overall Runtimes for Case Studies 1-4 using ATLANTIS.

Next, to present the feature-specific runtime profiles of ATLANTIS, we clocked step-wise runtimes for case studies 1-4 (see Supplementary Table S6). The features that were individually benchmarked included weights-based and rules-based DA, heuristic and exhaustive state space PA, trajectory mapping, landscape construction and visualization. Again, the three aforementioned hardware configurations were employed to clock each step. The compute times for weights-based DA clocked between 1-8 seconds, whereas the rules-based DA took between 1076-3440 seconds. Heuristic PA in case study 2 took less than one second while exhaustive PA of CS1 took 2-5 seconds. Exhaustive PA in CS2 could only be performed using high-memory Server configuration with compute time of 194 seconds. Data on feature-specific compute times has been provided in Supplementary Table S6.

After registering runtimes of various features offered by ATLANTIS, we compared them with three other similar tools, namely, BoolNet[12], BooleSim[13] and GINsim[16] using all three hardware configurations (see Supplementary Table S7). The benchmarking exercise was repeated for 11, 16 and 201-node networks to additionally gauge the impact of network sizes on runtimes. For DA of 11 nodes

network (case study 1), the runtimes of all tools were comparable (time < 1 seconds, see Supplementary Table S7a). This time slightly increased for ATLANTIS (from < 2 to 8 seconds) in case of 16 nodes network (case study 2) but remained mostly unchanged for other tools (see Supplementary Table S7b). However, for 201 nodes network (case studies 3 and 4), ATLANTIS successfully analyzed 10,000 randomly selected input states in 1380-3440 seconds for different hardware configurations. On the other hand, BooleSim[13] updated a single input state in approximately 10 seconds, while BoolNet[12] failed to perform the analysis. GINsim[16] runtimes could not be determined in this case as it does not directly admit rules-based regulation which was employed in case study 3 (see Supplementary Table S7c).

For exhaustive PA, ATLANTIS took around 2-5 seconds for 11 nodes (see Supplementary Table S7a). For 16 nodes, ATLANTIS required the Server configuration and returned the results in a maximum of 194 seconds (see Supplementary Table S7b). None of the other tools could undertake this exhaustive analysis. Heuristic PA runtimes were comparable for ATLANTIS and BoolNet[12] (between 1-2 and < 1 seconds, respectively). Attractor landscape construction cost was between 2-12 seconds using ATLANTIS. However, these runtimes could not be compared with BoolNet[12], BooleSim[13] and GINsim[16] due to unavailability of the feature in these tools. Heuristic PA runtimes were compared with BoolNet[12] only as BooleSim[13] and GINsim[16] did not offer this feature as well.

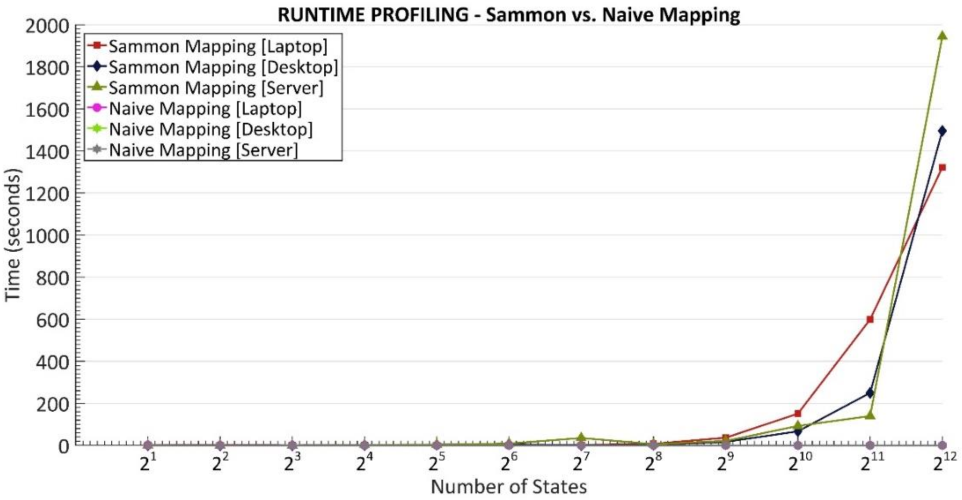

Supplementary Figure S16. Runtime comparison of Sammon and Naïve Transformation. A compute time vs. state size plot depicts the runtime cost incurred in both Sammon and Naïve mapping schemes.

Lastly, we also profiled the Sammon and Naïve projection strategies to provide runtime insights into the two strategies. Supplementary Figure S16 clearly shows that compute times for Sammon mapping rise exponentially, as the number of states increase. On the other hand, Naïve mapping performs linearly even for large state-spaces. The runtime profiles for different system configurations highlight the suitability of Naïve mapping for large state-spaces (see Supplementary Figure S16).

Individual Runtimes of Salient Features in ATLANTIS		Computational Time (*in seconds*)											
		Laptop Configuration (CPU: 2.50 GHz, RAM: 4 GB)				Desktop Configuration (CPU: 3.60 GHz, RAM: 32 GB)				Server Configuration (CPU: 2.40 GHz, RAM: 128 GB)			
		CS-1	CS-2	CS-3	CS-4	CS-1	CS-2	CS-3	CS-4	CS-1	CS-2	CS-3	CS-4
Type of Analysis	Weights-based Deterministic Analysis (DA)	< 1	≤ 8	–	–	< 1	≤ 2	–	–	< 1	≤ 6	–	–
	Rules-based Deterministic Analysis	–	–	≤ 3440	≤ 2615	–	–	≤ 1380	≤ 1076	–	–	≤ 2034	≤ 1572
	Save DA Results	< 1	4	≤ 75	≤ 47	< 1	2	≤ 30	≤ 19	< 1	2	≤ 40	≤ 26
	* Heuristic Probablistic Analysis (PA)	–	< 1	–	–	–	< 1	–	–		< 1	–	–
	Exhaustive Probablistic Analysis	5	FTC	–	–	2	FTC	–	–	4	≤ 194	–	–
	Trajectory Mapping	< 1	–	–	–	< 1	–	–	–	< 1	–	–	–
	Save PA Results	< 1	1	–	–	< 1	< 1	–	–	< 1	≤ 12	–	–
Visualization & Landscape Construction	Network Sketching Using Biograph	< 1	< 1	17	17	< 1	< 1	9	9	< 1	< 1	13	12
	Cell Fate Landscape Construction	–	< 1	≤ 1	≤ 1	–	< 1	< 1	< 1	–	< 1	< 1	< 1
	Attractor Landscape Construction	≤ 6	≤ 3	–	–	≤ 2	≤ 2	–	–	≤ 4	≤ 12	–	–

*Heuristically pruned state-space was employed in probablistic analysis

Note: For case studies 1 and 2, the "Simulation Timesteps" were set at 3000 and 17000, respectively. From 3 repeated runs, only the largest runtimes have been reported for ATLANTIS

FTC : Failed to compute

– : Not performed

Supplementary Table S6. Individual Runtimes of Salient Features in ATLANTIS.

(a) 11 Nodes Network[1] Runtime Comparison of ATLANTIS with Other tools		Computational Time (*in seconds*)											
		Laptop Configuration (CPU: 2.50 GHz, RAM: 4 GB)				Desktop Configuration (CPU: 3.60 GHz, RAM: 32 GB)				Server Configuration (CPU: 2.40 GHz, RAM: 128 GB)			
		ATLANTIS	BoolNet	BooleSim	GINsim	ATLANTIS	BoolNet	BooleSim	GINsim	ATLANTIS	BoolNet	BooleSim	GINsim
Type of Analysis	Deterministic Analysis	< 1	< 1	< 1	< 1	< 1	< 1	< 1	< 1	< 1	< 1	< 1	< 1
	Exhaustive Probablistic Analysis	5	n/a	n/a	n/a	2	n/a	n/a	n/a	4	n/a	n/a	n/a
	*Heuristic Probablistic Analysis	2	< 1	n/a	n/a	< 1	< 1	n/a	n/a	1	< 1	n/a	n/a
Visualization & Landscape Construction	Network Sketching	< 1	< 1	< 1	< 1	< 1	< 1	< 1	< 1	< 1	< 1	< 1	< 1
	Cell Fate Landscape Construction	< 1	n/a	n/a	n/a	< 1	n/a	n/a	n/a	< 1	n/a	n/a	n/a
	Attractor Landscape Construction	≤ 6	n/a	n/a	n/a	2	n/a	n/a	n/a	≤ 4	n/a	n/a	n/a

(b) 16 Nodes Network[2] Runtime Comparison of ATLANTIS with Other tools		Computational Time (*in seconds*)											
		Laptop Configuration (CPU: 2.50 GHz, RAM: 4 GB)				Desktop Configuration (CPU: 3.60 GHz, RAM: 32 GB)				Server Configuration (CPU: 2.40 GHz, RAM: 128 GB)			
		ATLANTIS	BoolNet	BooleSim	GINsim	ATLANTIS	BoolNet	BooleSim	GINsim	ATLANTIS	BoolNet	BooleSim	GINsim
Type of Analysis	Deterministic Analysis	≤ 8	< 1	< 1	< 2	≤ 2	< 1	< 1	< 2	≤ 6	< 1	< 1	< 2
	Exhaustive Probablistic Analysis	FTC	n/a	n/a	n/a	FTC	n/a	n/a	n/a	≤ 194	n/a	n/a	n/a
	*Heuristic Probablistic Analysis	< 1	< 1	n/a	n/a	< 1	< 1	n/a	n/a	< 1	< 1	n/a	n/a
Visualization & Landscape Construction	Network Sketching	< 1	< 1	< 1	< 1	< 1	< 1	< 1	< 1	< 1	< 1	< 1	< 1
	Cell Fate Landscape Construction	< 1	n/a	n/a	n/a	< 1	n/a	n/a	n/a	< 1	n/a	n/a	n/a
	Attractor Landscape Construction	≤ 3	n/a	n/a	n/a	2	n/a	n/a	n/a	≤ 12	n/a	n/a	n/a

(c) 201 Nodes Network[3] Runtime Comparison of ATLANTIS with Other tools		Computational Time (*in seconds*)											
		Laptop Configuration (CPU: 2.50 GHz, RAM: 4 GB)				Desktop Configuration (CPU: 3.60 GHz, RAM: 32 GB)				Server Configuration (CPU: 2.40 GHz, RAM: 128 GB)			
		ATLANTIS	BoolNet	BooleSim	GINsim	ATLANTIS	BoolNet	BooleSim	GINsim	ATLANTIS	BoolNet	BooleSim	GINsim
Type of Analysis	Deterministic Analysis	≤ 3440 †	FTC	10 ‡	n/a	≤ 1380 †	FTC	10 ‡	n/a	≤ 2034 †	FTC	10 ‡	n/a
Visualization & Landscape Construction	Network Sketching	17	< 1	< 1	< 1	9	< 1	< 1	< 1	13	< 1	< 1	< 1
	Cell Fate Landscape Construction	≤ 1	n/a	n/a	n/a	< 1	n/a	n/a	n/a	< 1	n/a	n/a	n/a

[1]Case study 1 network, adapted from Han *et al* , "Simulation Timesteps" set at 3000

[2]Case study 2 network, adapted from Choi *et al*, "Simulation Timesteps" set at 17000

[3]Case study 3 & 4 networks, adapted from Cho *et al*

*Heuristically pruned state-space was employed in probablistic analysis

† Rules-based deterministic analysis performed for 10,000 randomly selected states

‡ Rules-based deterministic analysis performed for a single state only (a limitation of BooleSim)

Note: All other parameters were kept at their default values. From 3 repeated runs, only the largest runtimes have been reported for ATLANTIS while for other tools, the smallest runtimes have been provided.

n/a : Feature not available

FTC : Failed to compute

– : Not performed

Supplementary Table S7. Effect of Network Size on Analysis Runtimes. Computational cost of analyzing (a) 11 nodes network, (b) 16 nodes network, and (c) 201 nodes network using ATLANTIS, BoolNet, BooleSim and GINsim.

E. Step by Step

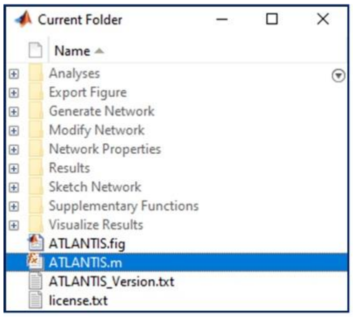

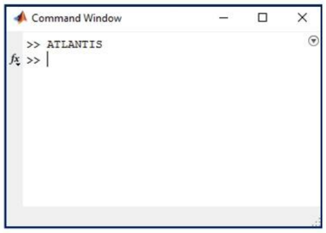

Type 'ATLANTIS' in the MATLAB Command Window

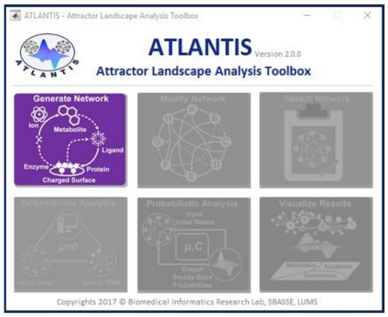

Step 1: Launch MATLAB and change the current working directory according to the path where ATLANTIS is downloaded.

Step 2: Type 'ATLANTIS' in the command window to start the toolbox.

Step 3: Main GUI of the toolbox pops up upon initializing the toolbox.

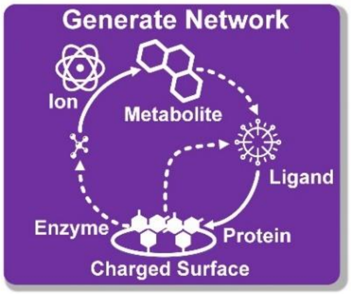

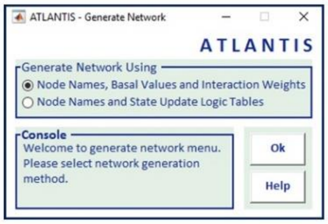

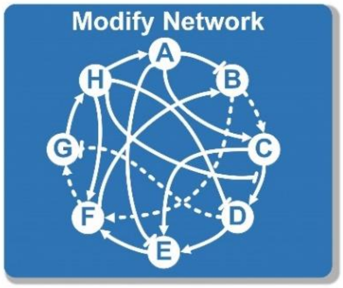

Step 4: Click 'Generate Network' to input a user defined network.

Step 5: Select the file containing the network description.

Step 6: Click 'Modify Network' to insert mutations in the network.

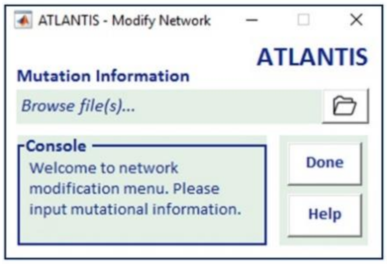

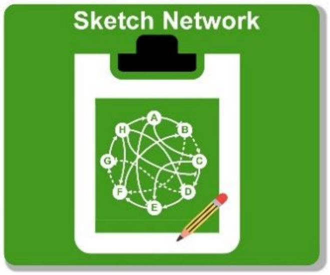

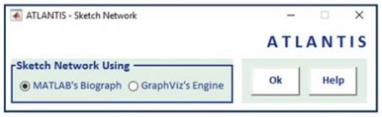

Step 7: Select the files listing node mutations and link modifications.

Step 8: Click 'Sketch Network' to visualize and assemble the network.

Step 9: Select 'Biograph' or 'Graphviz' for network visualization.

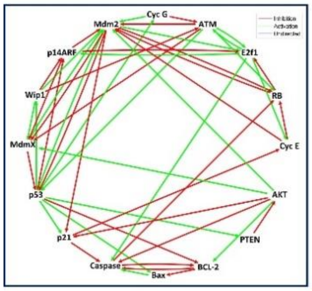

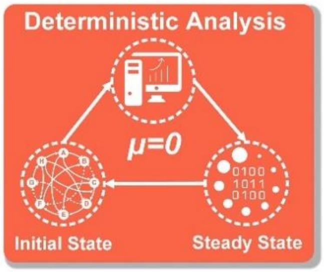

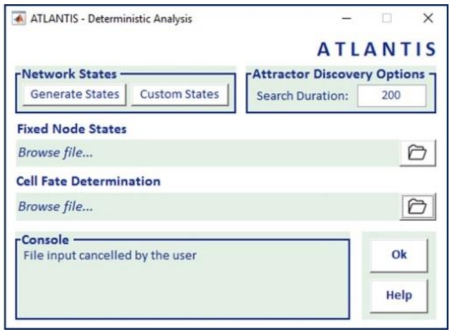

Step 10a: Graphviz outputs a network that resembles the one shown above.

Step 11a: Next click 'Deterministic Analysis' to perform the deterministic analysis (DA).

Step 12a: Enter input files for DA.

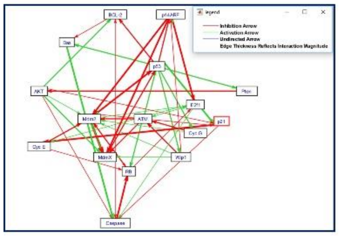
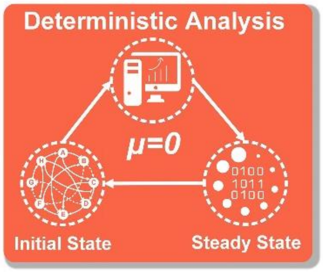

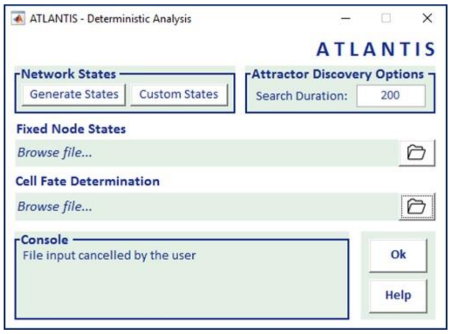

Step 10b: Biograph outputs a network that resembles the one shown above.

Step 11b: Next click 'Deterministic Analysis' to perform the DA.

Step 12b: Enter input files for DA.

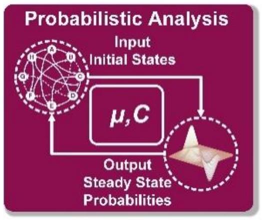

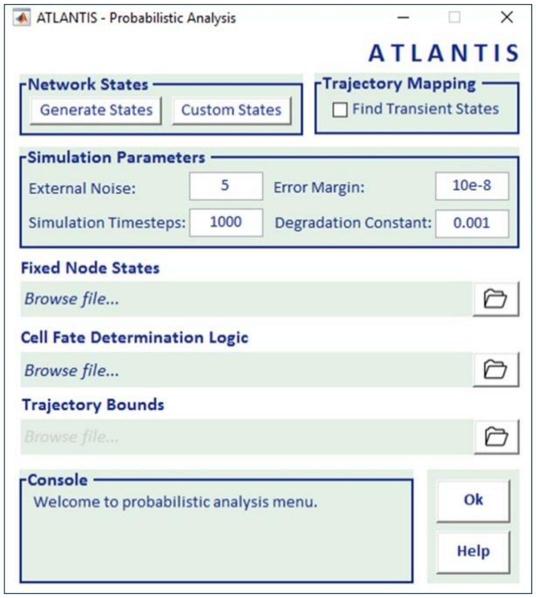

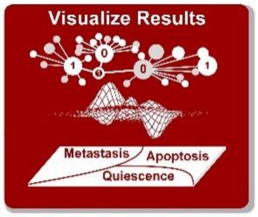

Step 13: Next click 'Probabilistic Analysis' to perform PA.

Step 14: Enter input files for PA.

Step 15: Click 'Visualize Results' to view results for the analyses.

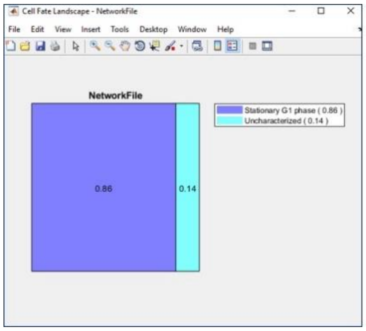

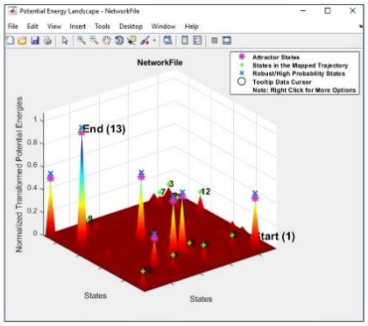

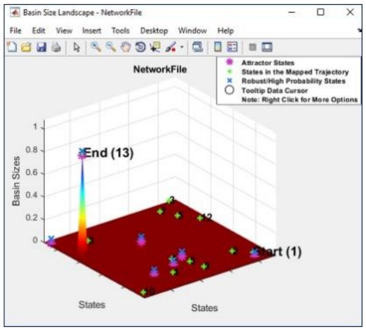

Step 16: Visualize Cell Fate Landscape (CFL) using ATLANTIS.

Step 17: Visualize Potential Energy Landscape (PEL) using ATLANTIS.

Step 19: Visualize Basin Size Landscape (BSL) using ATLANTIS.

F. Availability

Item	Description	Link
**Project Home**	URL link for ATLANTIS project home	https://github.com/BIRL/ATLANTIS
**Downloads**	Download ATLANTIS	https://github.com/BIRL/ATLANTIS/archive/master.zip
**Graphviz**	Downloadable version of Graphviz	https://github.com/BIRL/ATLANTIS/tree/master/GraphViz%20-%20Setup
**User's Manual**	ATLANTIS User manual can be found here.	https://github.com/BIRL/ATLANTIS/tree/master/User%20Manual
**Pull Requests**	Pull Requests page is available at this link where codes for ATLANTIS are collaborated.	https://github.com/BIRL/ATLANTIS/pulls
**Issues**	Issues and related discussions can be made at the issues page for ATLANTIS.	https://github.com/BIRL/ATLANTIS/issues
**Datasets**	Sample Data files are available at the following link, for ATLANTIS.	https://github.com/BIRL/ATLANTIS/tree/master/Sample%20Data
**ATLANTIS Executable**	Public Mega folder containing the ATLANTIS Executable file (ATLANTIS.exe) for 64-bit Windows® operating system	https://mega.nz/#F!x7wRFDZY!t2PQTdZZawF_Wiqn5rfepw

G. Video Tutorials

Video	Link
**Complete Playlist** - The following videos have been made available in the form of a playlist "ATLANTIS v2.0.0" at the following link.	https://www.youtube.com/watch?v=oNTgIW4-dd4&list=PLaNVq-kFOn0YD_4lfJEEEzISNuqnTZVIa
**Launch ATLANTIS**	https://www.youtube.com/watch?v=oNTgIW4-dd4
**Setting Up Graphviz for ATLANTIS**	https://www.youtube.com/watch?v=l6MbaJ9MMRA
**Decoding Yeast Cell Cycle Progression - Case Study 1**	https://www.youtube.com/watch?v=ySvGu2b5H2c
**Evaluation of Combinatorial Therapeutic Efficacy - Case Study 2**	https://www.youtube.com/watch?v=9t15WC5NCek
**Temporal Evolution of Cell Fate Landscape during Colorectal Tumorigenesis - Case Study 3**	https://www.youtube.com/watch?v=CFUtIyf5XcA
**In silico Drug Screening in ATLANTIS - Case Study 4**	https://www.youtube.com/watch?v=uXaC_2X36uA
**Deciphering the Transient Biological States by Trajectory Mapping in ATLANTIS**	https://www.youtube.com/watch?v=r81u_7Ku3e0
**Interactively visualize Attractor Landscapes - Rotate, Invert, Adjust Transparency**	https://www.youtube.com/watch?v=dyI-vDXJj3I
**Visualize Attractors in Basin Size Landscape**	https://www.youtube.com/watch?v=mH29IKbbyq8

**Interactive Visualization of Biomolecular Networks**	https://www.youtube.com/watch?v=Y6K4ETHD4GQ
**Cancel Simulation in ATLANTIS**	https://www.youtube.com/watch?v=Dan_SdgSh5U
**Viewing and Reusing Network State List files in '.csv' and '.mat' Format**	https://www.youtube.com/watch?v=07NAysmTjiQ
**Build and Install ATLANTIS.exe**	https://www.youtube.com/watch?v=0lFV993ih-s
**Visualizing detailed information of Attractors and the states present in the mapped Trajectory**	https://www.youtube.com/watch?v=VoSCySbly3Q

H.Worked Examples

To facilitate the ease of use, we present several elaborate worked out examples using ATLANTIS.

1. Decoding Yeast Cell Cycle Progression using Attractor Landscape Analysis

Step-1. Click the 'Generate Network' button, select the option with 'Node Names, Basal Values and Interaction Weights'. Next, click 'Browse file' to select the network. The network file for yeast cell cycle network (case study 1) can be found at: ATLANTIS\Sample Data\Case Study 1\Input Files\NetworkFile.txt

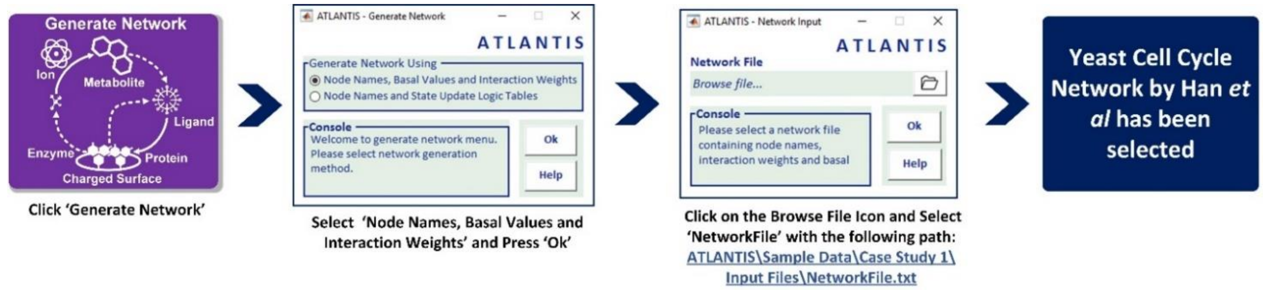

Step-2. After loading the network information file, click on 'Sketch Network' button. Select Graphviz or Biograph to visualize the specified Network. You can export the generated figure by right clicking on the figure.

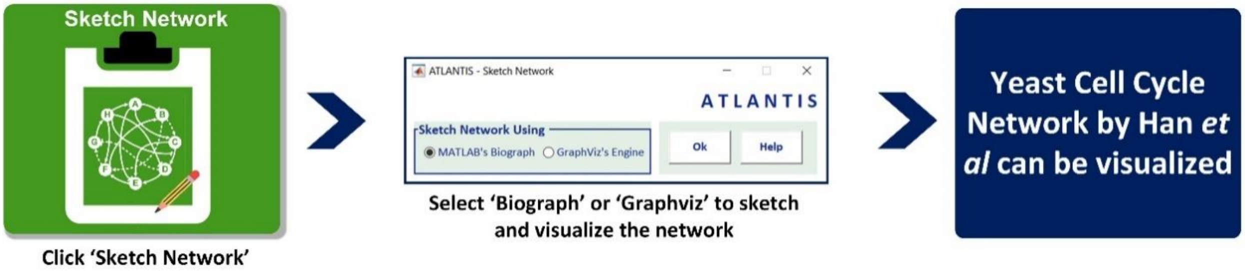

Step-3. After visualizing the network, click on the 'Deterministic Analysis' button. This would open the DA menu. The first step, now, would be generating the network states. Click on 'Generate States', select 'Exhaustive (Complete State Space)' option and press 'Ok'. After this step, move back to the DA Window, click on the 'Browse file' icon under 'Cell Fate Determination' and select the folder with the following path: ATLANTIS\Sample Data\Case Study 1\Input Files\Cell Fate Determination Logic.csv

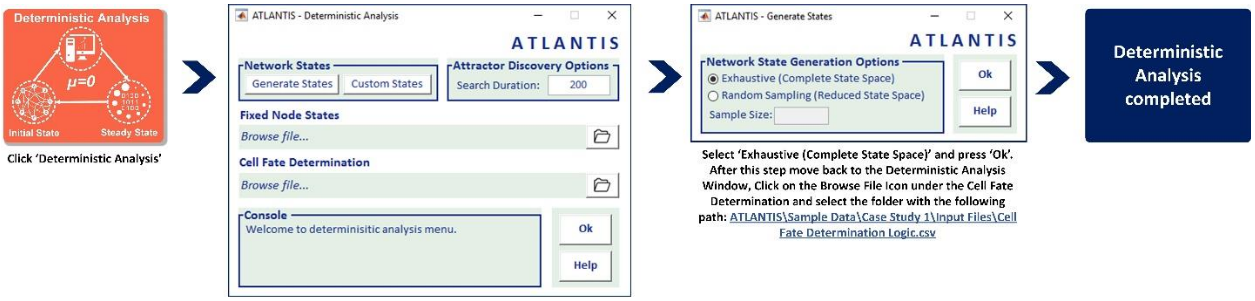

Step-4. After performing DA, the next step is to visualize the results. Click on the 'Visualize Results' button. Select 'CFL' and 'BSL' from the 'Plot Selection' pane. Specify the 'Treemap Plot Options' as shown in figure. Select 'Naïve Random' from the 'Mapping Method' pane, specify 'Attractor Plot Options' and click 'Plot'. You can export the generated figures as well.

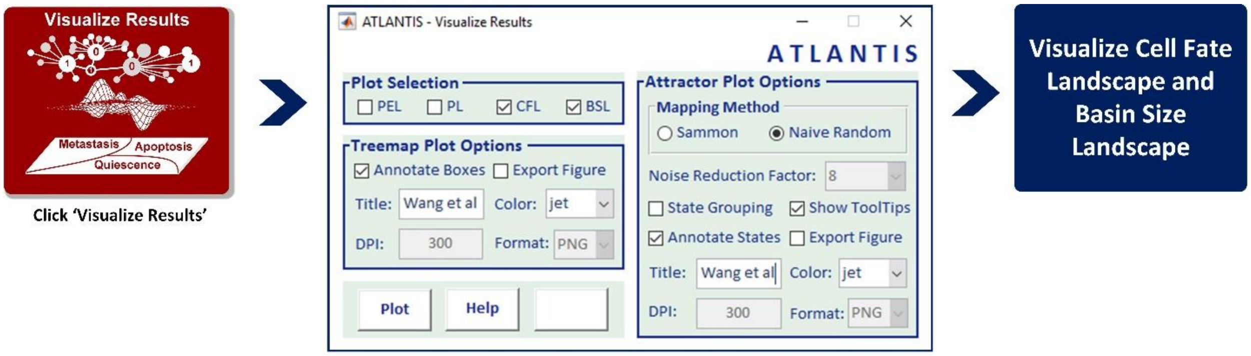

Select 'CFL' and 'BSL' from Plot Selection Options. Specify the Treemap Plot Options. Select 'Naïve Random' from Mapping Methods and specify Attractor Plot Options and press 'Plot'

Step-5. In order to perform PA, click on 'Probabilistic Analysis' button. Click on 'Generate States', if no modification is required in the network Select 'Find Transient States' from the 'Trajectory Mapping' pane. Click on the 'Browse file' option under 'Trajectory Bounds' and select file with the following path: ATLANTIS\Sample Data\Case Study 1\Input Files\TrajectoryBounds.csv

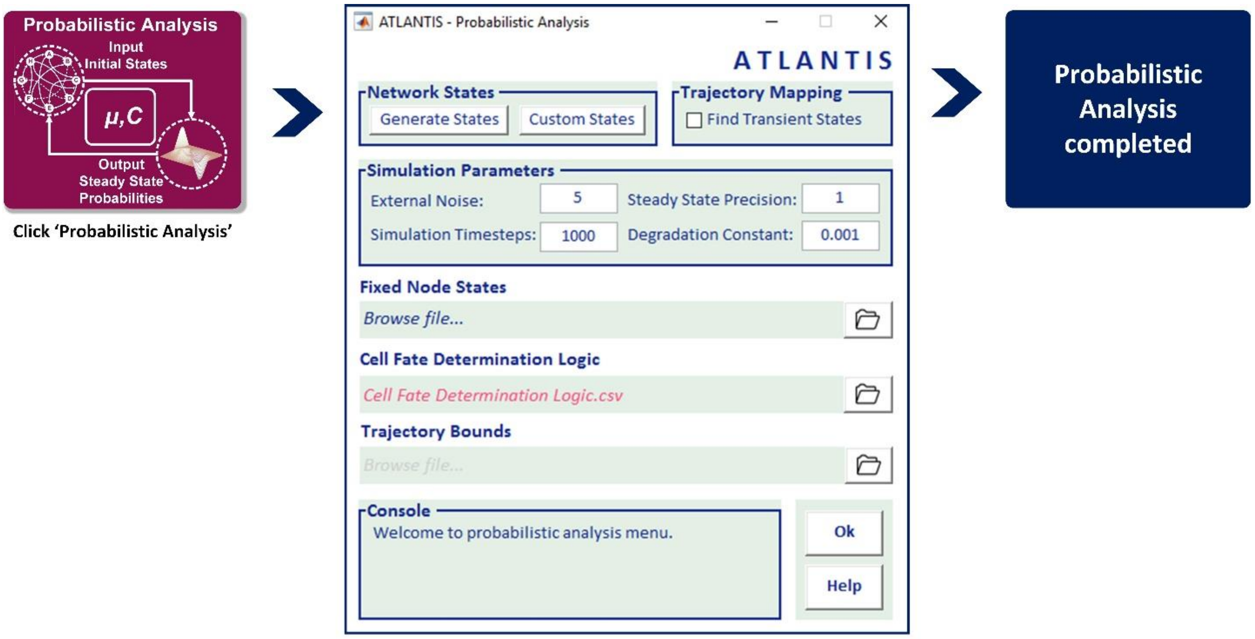

Click on 'Generate States' , if no modification is required in the network Select 'Find Transient States' from Trajectory Mapping. Click on the Browse file option under Trajectory Bounds and select file with the following path:ATLANTIS\Sample Data\Case Study 1\Input Files\TrajectoryBounds.csv

Step-6. After performing the PA, the next step is to visualize the results. Click on the 'Visualize Results' button. Select 'PEL' and 'PL' from 'Plot Selection Options'. Select "Naïve Random" from 'Mapping Method' and specify 'Attractor Plot Options' and press 'Plot'. You can export the generated figures as well.

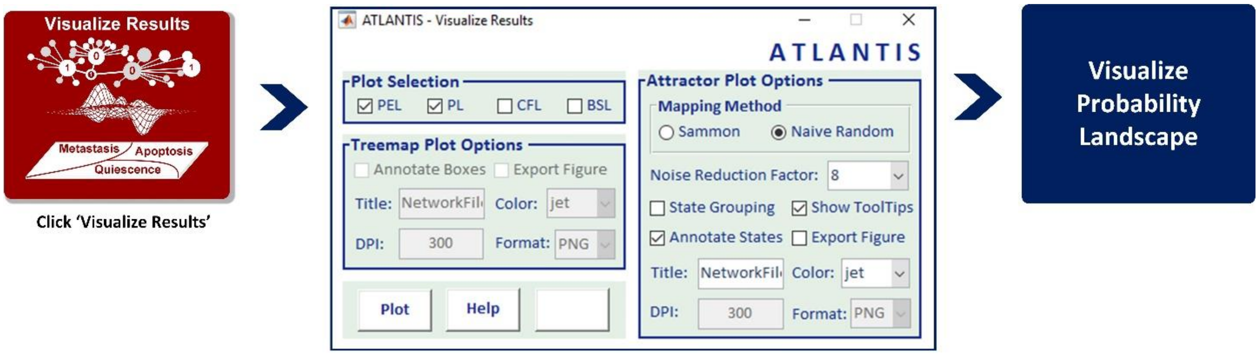

Select 'PEL' and 'PL' from Plot Selection Options. Select 'Naïve Random' from Mapping Methods and specify Attractor Plot Options and press 'Plot'

2. Investigating the Evolution of Cell Fate Landscape during Colorectal Tumorigenesis

Step-1. After clicking the 'Generate Network' button, select the option with 'Node Names and State Update Logic Tables'. Next, browse the file and folder at given link: ATLANTIS\Sample Data\Case Study 3\Input Files\Network Details for further analysis.

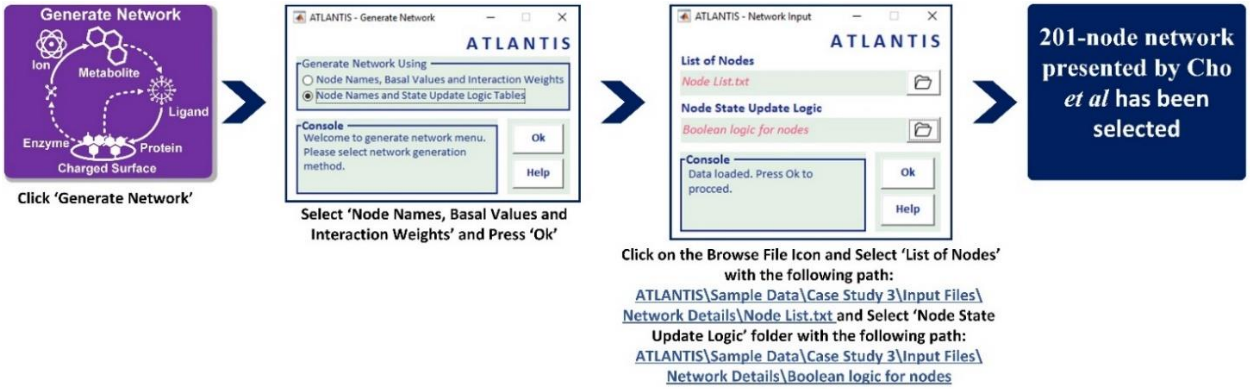

Step-2. Click on 'Deterministic Analysis' button. This would open the DA menu. The first step, now, would be generating the network state-space. Click on 'Custom States' and upload the file containing custom network states. The path of the file is given as: ATLANTIS\Sample Data\Case Study 3\Input Files\Network State Lists\Simulation 1

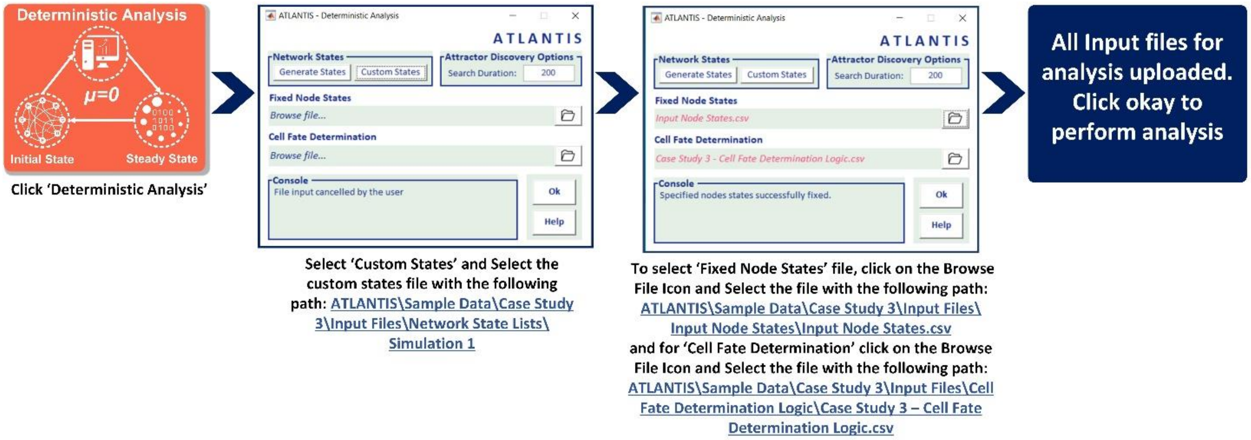

Step-3. On completion of DA move on to visualizing the results. Here, to visualize the Treemap plot for phenotype classification, check 'CFL', enter a title for your plot and then click 'Plot'.

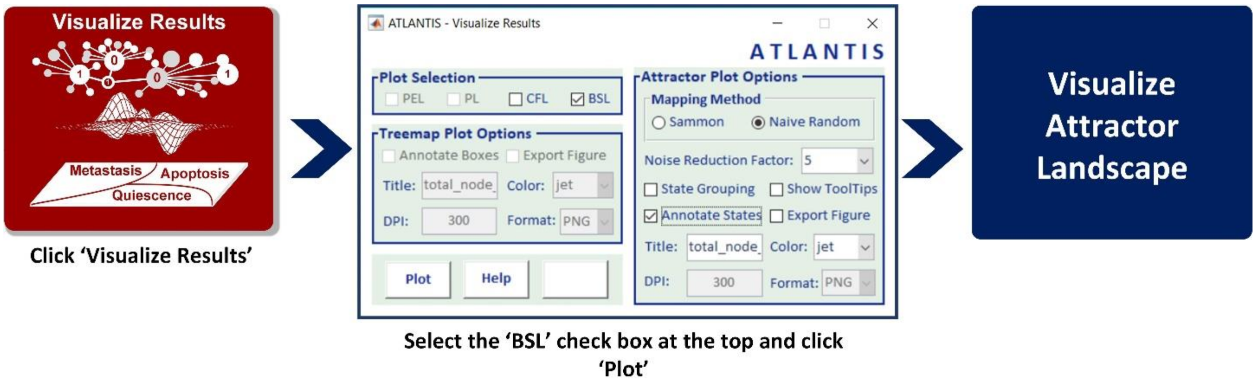

Step-4. To visualize the attractor landscape, check 'BSL', enter remaining parameters and click 'Plot'.

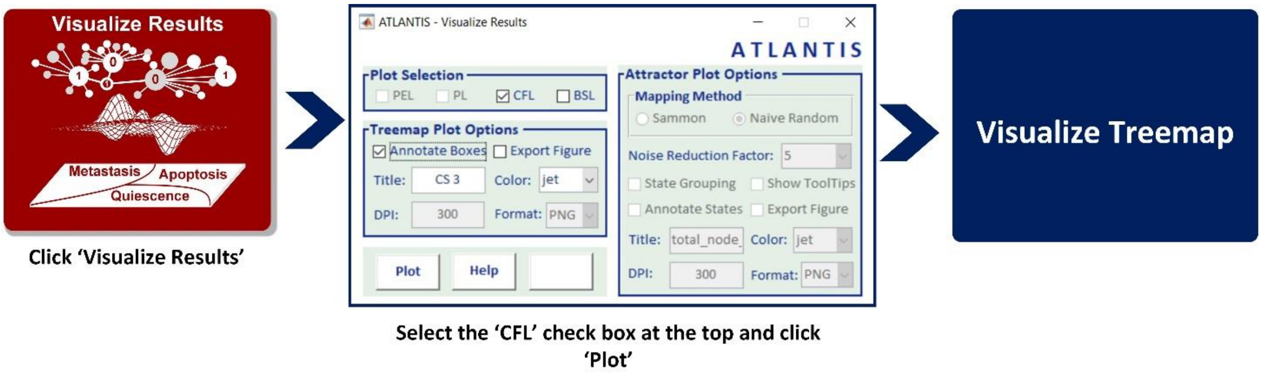

3. Demonstrating the use of 'Trajectory Mapping' in Yeast Cell Cycle Progression Network

Step-1. Create 'Trajectory Bounds.csv' file for use in Step-4. The first and second row in the file represents the initial and final boundary state for complete trajectory respectively.

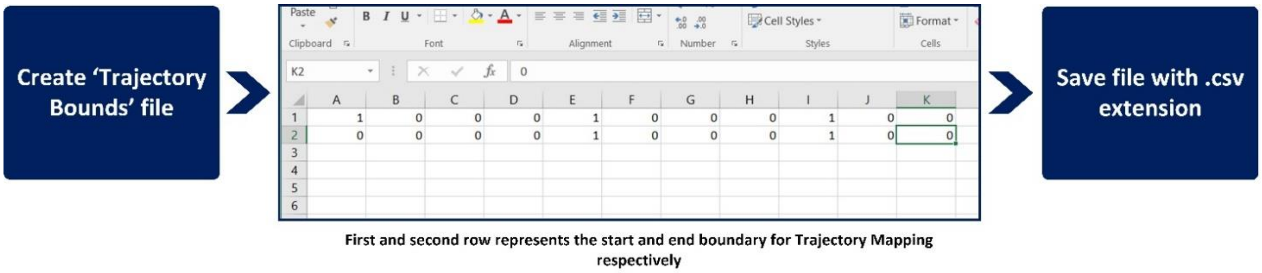

Step-2. Click the 'Generate Network' button, select the option with 'Node Names, Basal Values and Interaction Weights'. Next, browse the file at given link for further analysis. The network file for yeast cell cycle can be found at: ATLANTIS\Sample Data\Case Study 1\Input Files\NetworkFile.txt

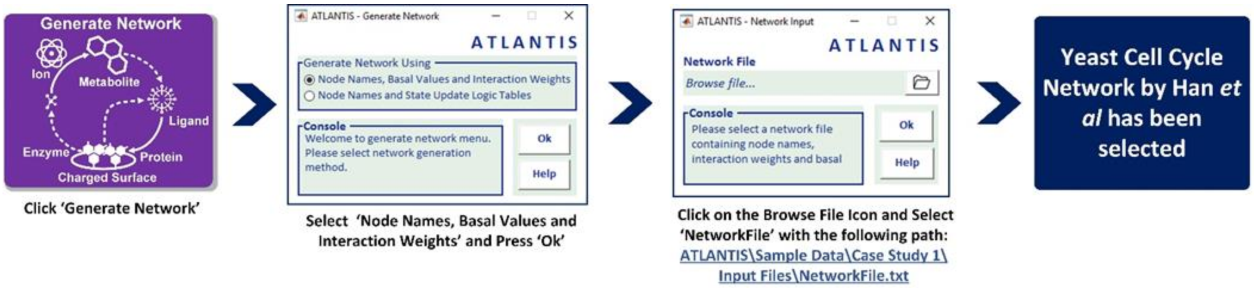

Step-3. After generating the network, click on 'Deterministic Analysis' button. This would open the DA menu. The first step, now, would be generating the network states. Click on 'Generate States' and select 'Exhaustive (Complete State Space)' and press 'Ok'. After this step, move back to the

DA window, click on the 'Browse file' icon under 'Cell Fate Determination' and select the folder with the following path: ATLANTIS\Sample Data\Case Study 1\Input Files\Cell Fate Determination Logic.csv

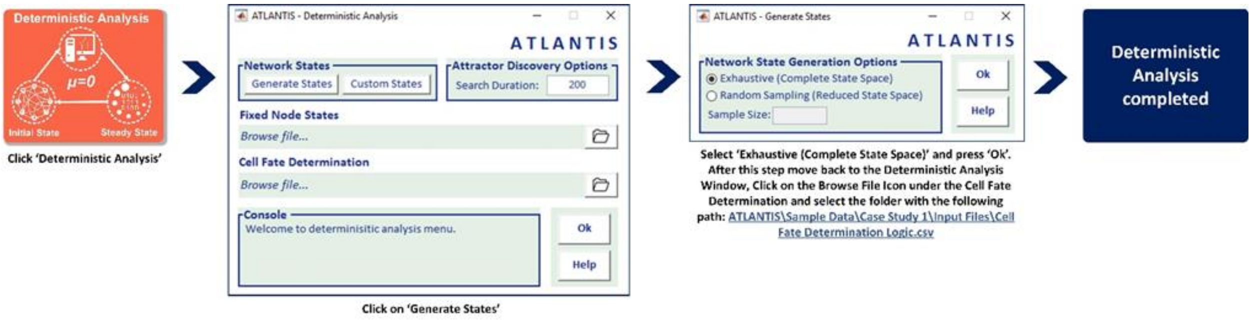

Step-4. Next to perform PA, click on 'Probabilistic Analysis' button. Click on 'Generate States' followed by exhaustive sampling of the initial state-space. Under 'Trajectory Mapping', select 'Find Transient States' checkbox. Click on the 'Browse file' option under 'Trajectory Bounds' and select file stored in Step-1.

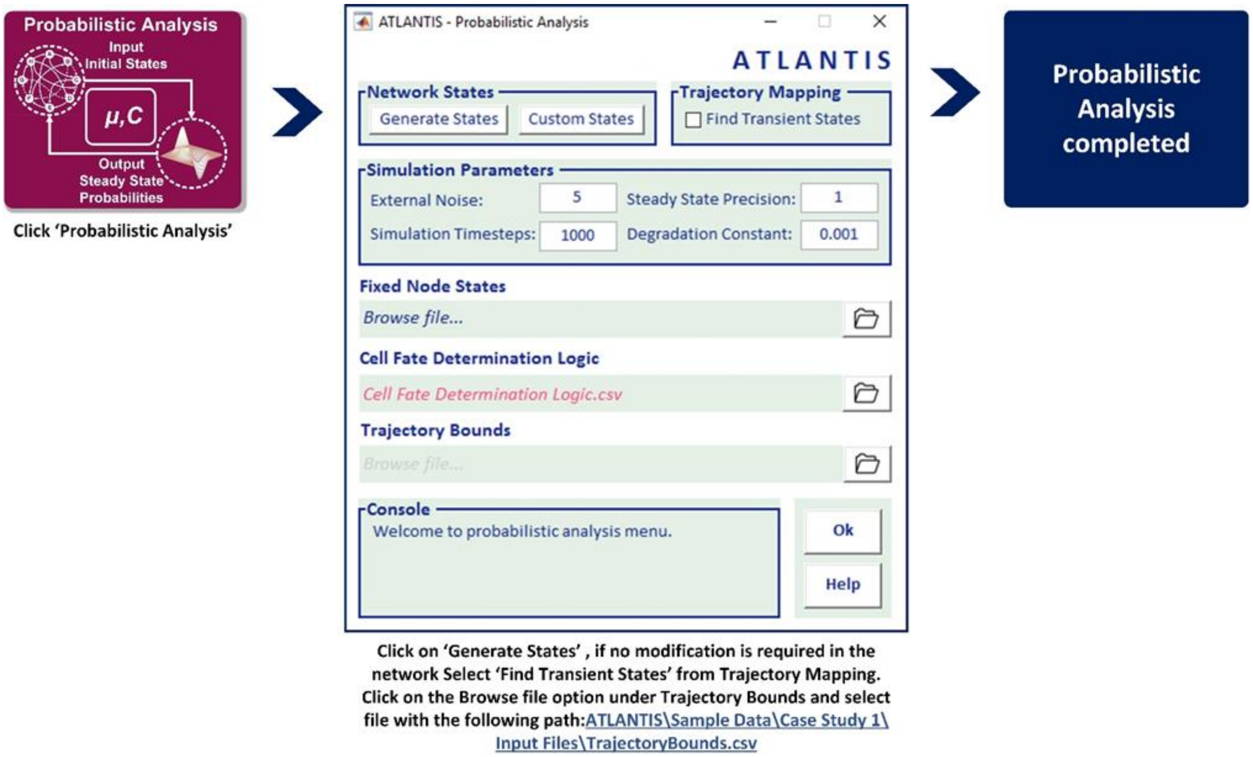

Step-5. After performing the analyses, the next step is to visualize the results. Click on the 'Visualize Results' button. Select 'PEL' and 'PL' from 'Plot Selection' options. Select 'Naïve Random' from Mapping Methods and check annotation and tooltips options and press 'Plot'.

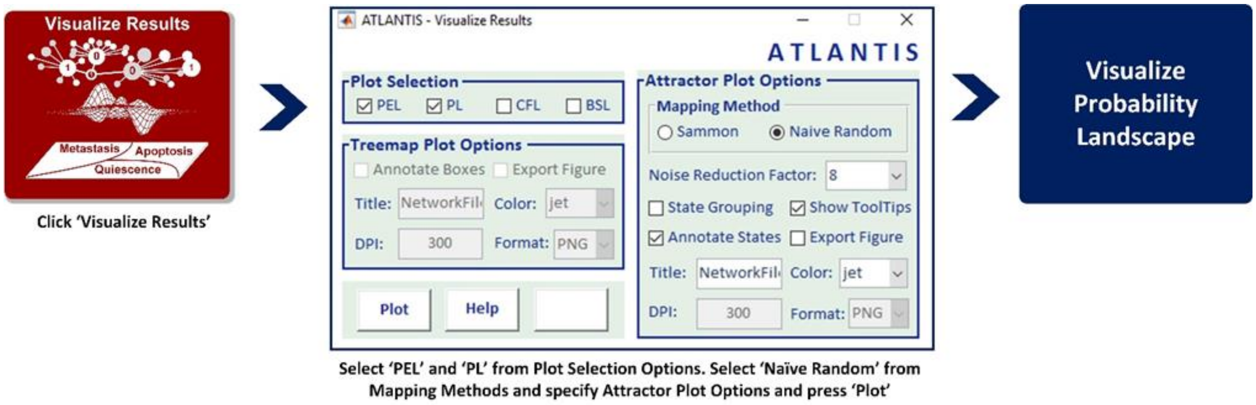

Step-6. To display transient states trajectory with in the attractor landscape, right-click on the figure, click 'Toggle Annotations' and then click 'Show Arrows Linking States in Trajectory'.

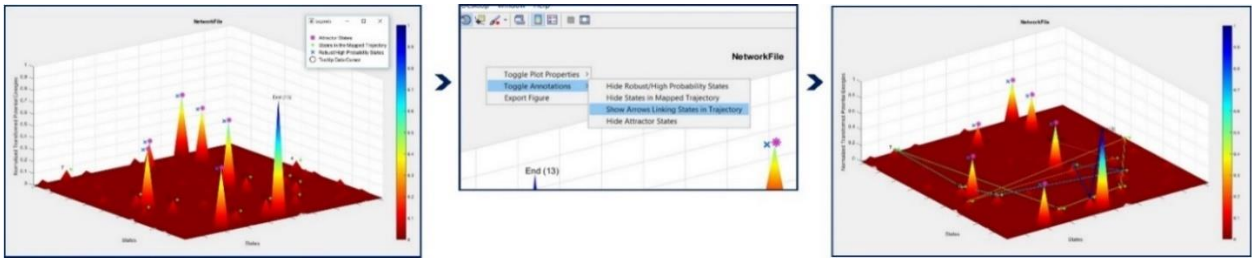

Step-7. To view the trajectory path alone, right-click on the screen, click 'Toggle Plot Properties' and then click 'Hide Surface Plot'.

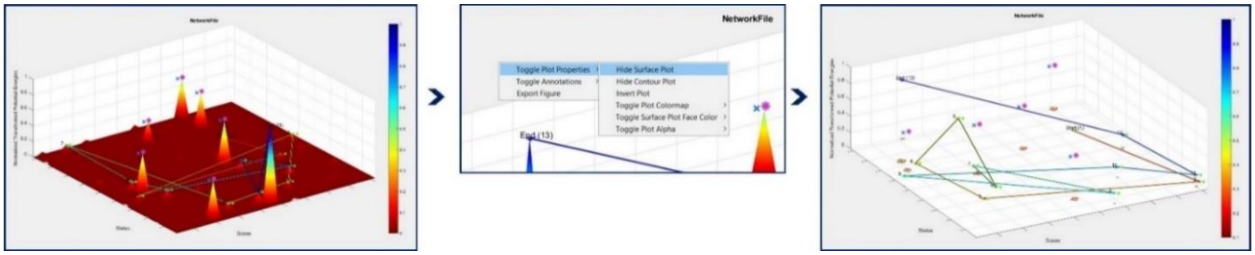

Step-8. A variety of visualization options can be used to clearly view trajectory path and transient states. The trajectory path traces the route from the initial boundary state to the final boundary state, passing through all the intermediate transient states. The color intensity at every point along the path corresponds to its probability.

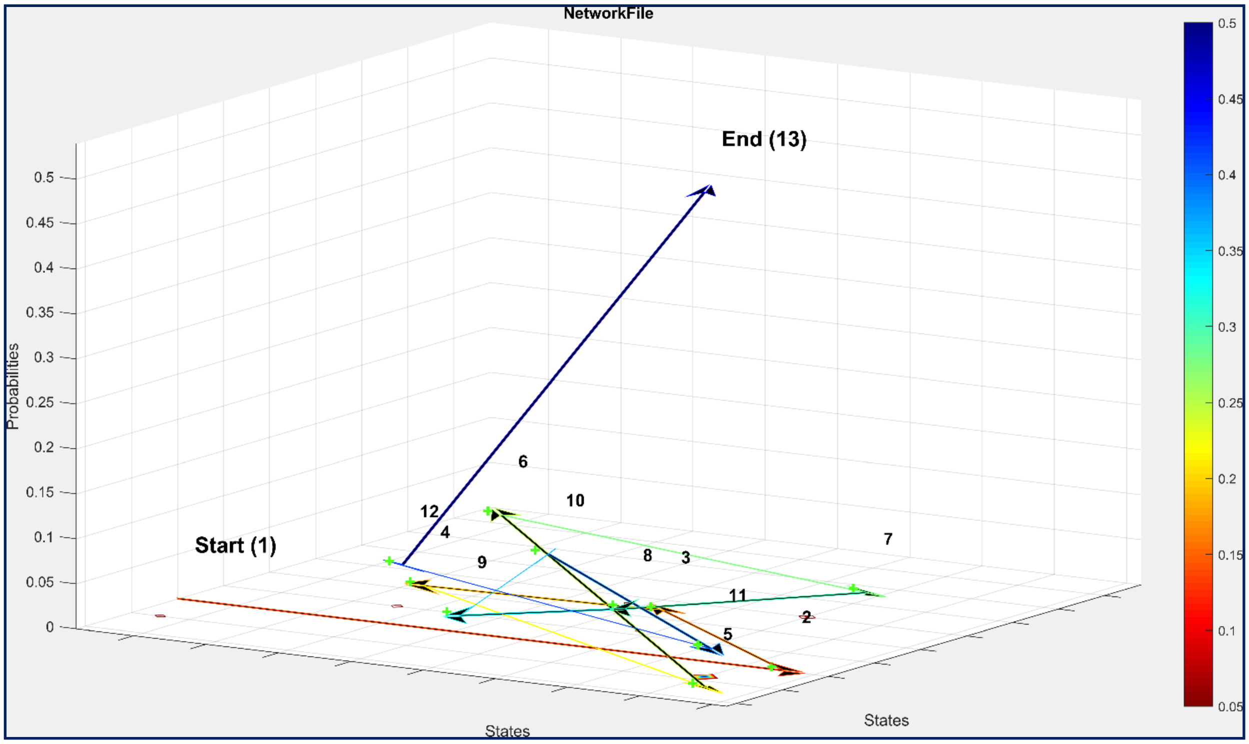

4. Demonstrating the use of ‘Fixed Nodes States’ feature in 201-node network for Colorectal Tumorigenesis

Step-1. Create ‘Input Node States.csv’ file for use in Step-4. The first row shows the names of the input nodes to be updated and the second row shows their fixed values. Once fixed, the values for these input nodes will remain constant throughout the simulation.

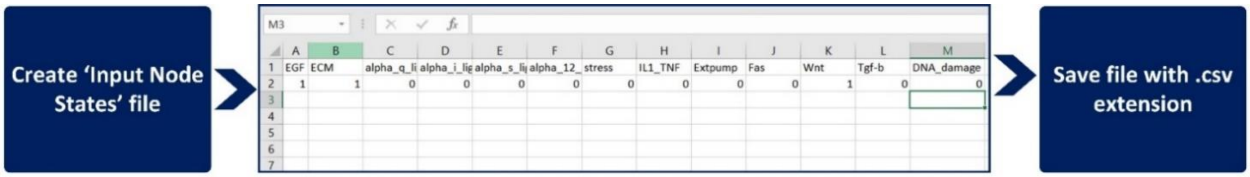

The first row shows the names of the Input Node to be updated and the second row shows their updated values

Step-2. The network file and update logic folder for colorectal tumorigenesis network can be found at: ATLANTIS\Sample_Data\Case_Study_3\Input_Files\Network_Details. After clicking the ‘Generate Network’ button, select the option with ‘Node Names and State Update Logic Tables’. Next, browse the file and folder at given link for further analysis.

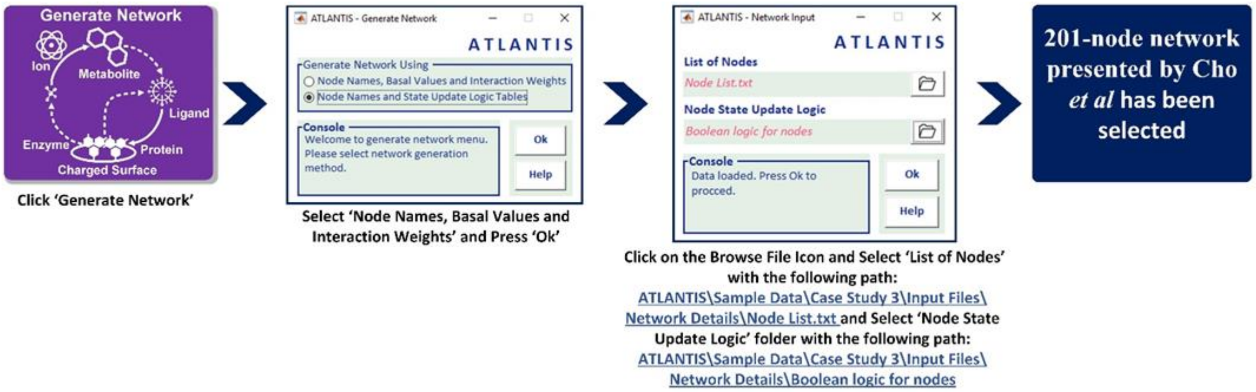

Step-3. Click on 'Deterministic Analysis' button. This would open the DA menu. The first step, now, would be generating the network state-space. Click on 'Custom States' and upload the file containing custom network states. The path of the file is given as: ATLANTIS\Sample Data\Case Study 3\Input Files\Network State Lists\Simulation 1

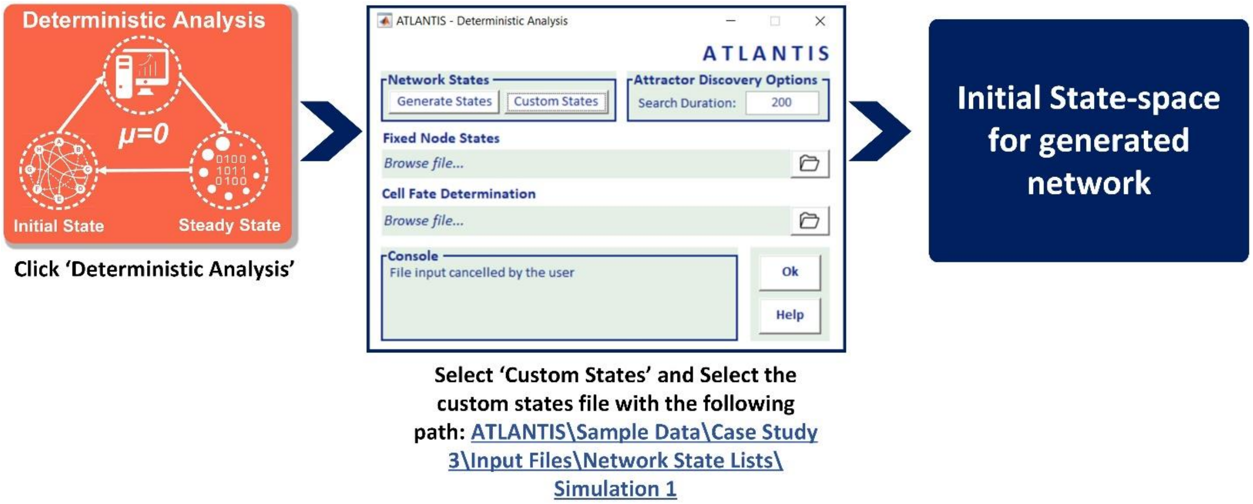

Step-4. To set the values of input nodes in a modular network, select the 'Fixed Node States' file. Click on the 'Browse file' option under 'Fixed Node States' and select file stored in Step-1.

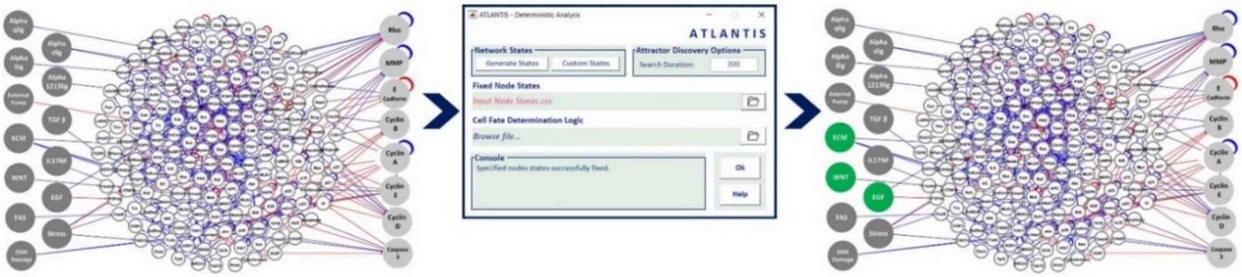

Step-5. Click 'Ok' on the DA menu to perform onward analysis.

5. Demonstrating the use of 'Custom States' feature in PA of Yeast Cell Cycle Progression Network

Step-1. Create 'Custom States file.csv' file for use in Step-3. Each row in the file represents a network state and each column represents a node's value in each state.

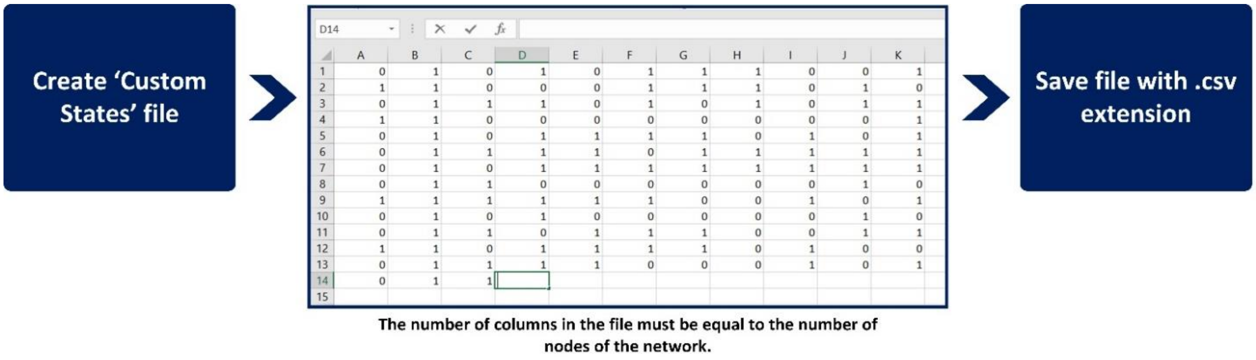

Step-2. Click the 'Generate Network' button, select the option with 'Node Names, Basal Values and Interaction Weights'. Next, browse the file at given link for further analysis. The network file for yeast cell cycle network can be found at: [ATLANTIS\Sample Data\Case Study 1\Input Files\NetworkFile.txt](ATLANTIS\Sample Data\Case Study 1\Input Files\NetworkFile.txt) .

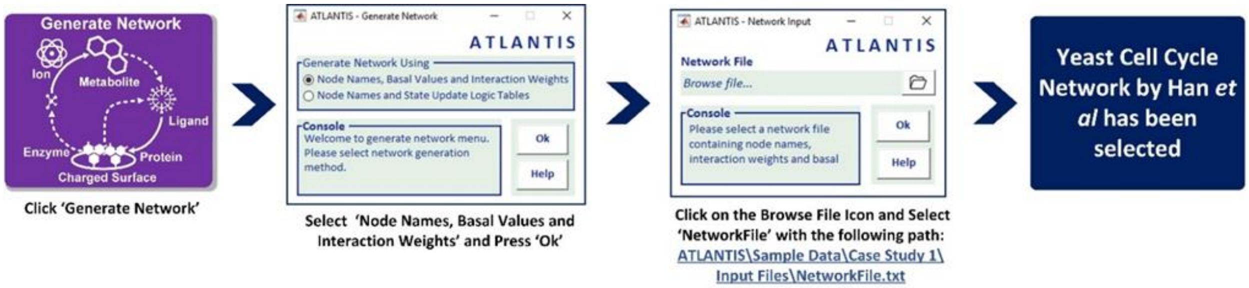

Step-3. Next to perform PA, click on 'Probabilistic Analysis' button. Click on 'Custom States' to upload the custom states file created in Step-1. Then click 'Ok' to perform analysis.

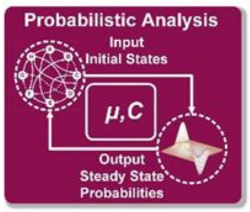

Click 'Probabilistic Analysis'

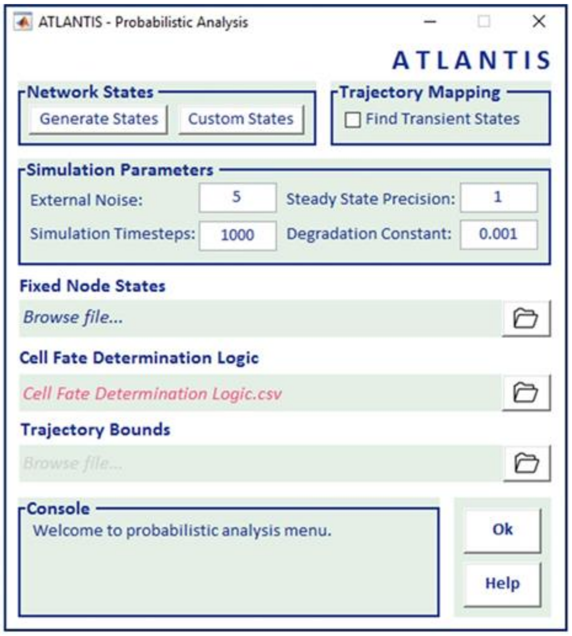

Click on 'Custom States' to upload the custom file created in Step-1

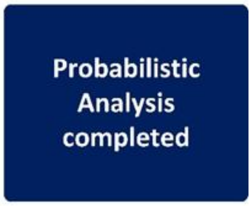

Supplementary References

1. Ho, D. Notepad++. (2016). Available at: https://notepad-plus-plus.org/.

2. Sublime Text. (2017). Available at: https://www.sublimetext.com/.

3. Choi, M., Shi, J., Jung, S. H., Chen, X. & Cho, K.-H. Attractor landscape analysis reveals feedback loops in the p53 network that control the cellular response to DNA damage. *Sci Signal* **5,** 83 (2012).

4. Kauffman, S., Peterson, C., Samuelsson, B. & Troein, C. Random Boolean network models and the yeast transcriptional network. *Proc. Natl. Acad. Sci.* **100,** 14796–14799 (2003).

5. Kauffman, S. A. Metabolic stability and epigenesis in randomly constructed genetic nets. *J. Theor. Biol.* **22,** 437–467 (1969).

6. Shmulevich, I., Dougherty, E. R., Kim, S. & Zhang, W. Probabilistic Boolean networks: a rule-based uncertainty model for gene regulatory networks. *Bioinformatics* **18,** 261–274 (2002).

7. Xiao, Y. A tutorial on analysis and simulation of boolean gene regulatory network models. *Curr. Genomics* **10,** 511–525 (2009).

8. Sammon, J. W. A Nonlinear Mapping for Data Structure Analysis. *IEEE Transactions on Computers* **C-18,** 401–409 (1969).

9. Cho, S.-H., Park, S.-M., Lee, H.-S., Lee, H.-Y. & Cho, K.-H. Attractor landscape analysis of colorectal tumorigenesis and its reversion. *BMC Syst. Biol.* **10,** 96 (2016).

10. Bamford, S. *et al.* The COSMIC (Catalogue of Somatic Mutations in Cancer) database and website. *Br. J. Cancer* **91,** 355–358 (2004).

11. Han, B. & Wang, J. Quantifying robustness and dissipation cost of yeast cell cycle network: the funneled energy landscape perspectives. *Biophys. J.* **92,** 3755–3763 (2007).

12. Müssel, C., Hopfensitz, M. & Kestler, H. A. BoolNet—an R package for generation, reconstruction and analysis of Boolean networks. *Bioinformatics* **26,** 1378–1380 (2010).

13. Bock, M., Scharp, T., Talnikar, C. & Klipp, E. BooleSim: An interactive Boolean network simulator. *Bioinformatics* **30,** 131–132 (2014).

14. Helikar, T. *et al.* The Cell Collective: Toward an open and collaborative approach to systems biology. *BMC Syst. Biol.* **6,** 96 (2012).

15. Klamt, S., Saez-Rodriguez, J. & Gilles, E. D. Structural and functional analysis of cellular networks with CellNetAnalyzer. *BMC Syst. Biol.* **1,** 2 (2007).

16. Gonzalez, A. G. *et al.* GINsim: a software suite for the qualitative modelling, simulation and analysis of regulatory networks. *Biosystems* **84,** 91–100 (2006).

17. Schwab, J., Burkovski, A., Siegle, L., Müssel, C. & Kestler, H. A. ViSiBooL-visualization and simulation of Boolean networks with temporal constraints. *Bioinformatics* **33,** 601–604 (2017).

18. Paroni, A. *et al.* CABeRNET: a Cytoscape app for augmented Boolean models of gene regulatory NETworks. *BMC Bioinformatics* **17,** 64 (2016).
